# Vulnerability of Arctic Ocean microbial eukaryotes to sea ice loss

**DOI:** 10.1038/s41598-024-77821-9

**Published:** 2024-11-21

**Authors:** Victoria L. N. Jackson, Thomas Grevesse, Estelle S. Kilias, Deo F. L. Onda, Kirsten F. Young, Michael J. Allen, David A. Walsh, Connie Lovejoy, Adam Monier

**Affiliations:** 1 Living Systems Institute, University of Exeter, Exeter, EX4 4QD UK; 2 Biology Department, Concordia University, Montréal, QC H4B 1R6 Canada; 3 The Marine Science Institute, University of the Philippines, Manila, Philippines; 4 Biosciences, University of Exeter, Exeter, EX4 4QD UK; 5 Institut de Biologie Intégrative et des Systèmes (IBIS), Département de Biologie, Université Laval, Québec, QC G1V 0A6 Canada; 6 Present Address: Department of Biology, University of Oxford, Oxford, OX1 3SZ UK

**Keywords:** Microbial ecology, Climate-change ecology, Molecular ecology, Microbial biooceanography

## Abstract

The Arctic Ocean (AO) is changing at an unprecedented rate, with ongoing sea ice loss, warming and freshening impacting the extent and duration of primary productivity over summer months. Surface microbial eukaryotes are vulnerable to such changes, but basic knowledge of the spatial variability of surface communities is limited. Here, we sampled microbial eukaryotes in surface waters of the Beaufort Sea from four contrasting environments: the Canada Basin (open ocean), the Mackenzie Trough (river-influenced), the Nuvuk region (coastal) and the under-ice system of the Canada Basin. Microbial community structure and composition varied significantly among the systems, with the most phylogenetically diverse communities being found in the more coastal systems. Further analysis of environmental factors showed potential vulnerability to change in the most specialised community, which was found in the samples taken in water immediately beneath the sea ice, and where the community was distinguished by rare species. In the context of ongoing sea ice loss, specialised ice-associated microbial assemblages may transition towards more generalist assemblages, with implications for the eventual loss of biodiversity and associated ecosystem function in the Arctic Ocean.

## Introduction

Environmental perturbations induced by anthropogenic activities are impacting the Arctic Ocean (AO) at a faster rate than other regions of the planet^1,2^. Mean air temperatures in the Arctic have risen at least twice as much as the global average in the past 20 years^3,4^. This warming is causing sea surface temperatures to rise, leading directly to the loss of older sea ice through increased melting^5^. The melting of thicker ice is transitioning the AO from a system dominated by multi-year ice (MYI) to one comprising primarily first-year ice (FYI)^6^, with both sea ice extent and thickness decreasing^7–9^. These changes have been particularly apparent in the Pacific-influenced Western AO^10^, where there have been marked reductions in sea ice cover^11^ and increased freshwater inflow from rivers due to a faster hydrological cycle and permafrost thawing in the High North^12–14^. The growing volume of terrestrial sources of freshwater flowing into the AO contributes to the ongoing freshening and heightened salinity stratification of the surface AO^15^, and leads to greater suspended sediment loads in surface waters^16^. These ongoing environmental perturbations are causing a regime shift towards a warmer, wetter and more unstable climate over the AO^17^, which is already impacting its biogeochemistry and ecosystem productivity^18–20^.

**Fig. 1:**
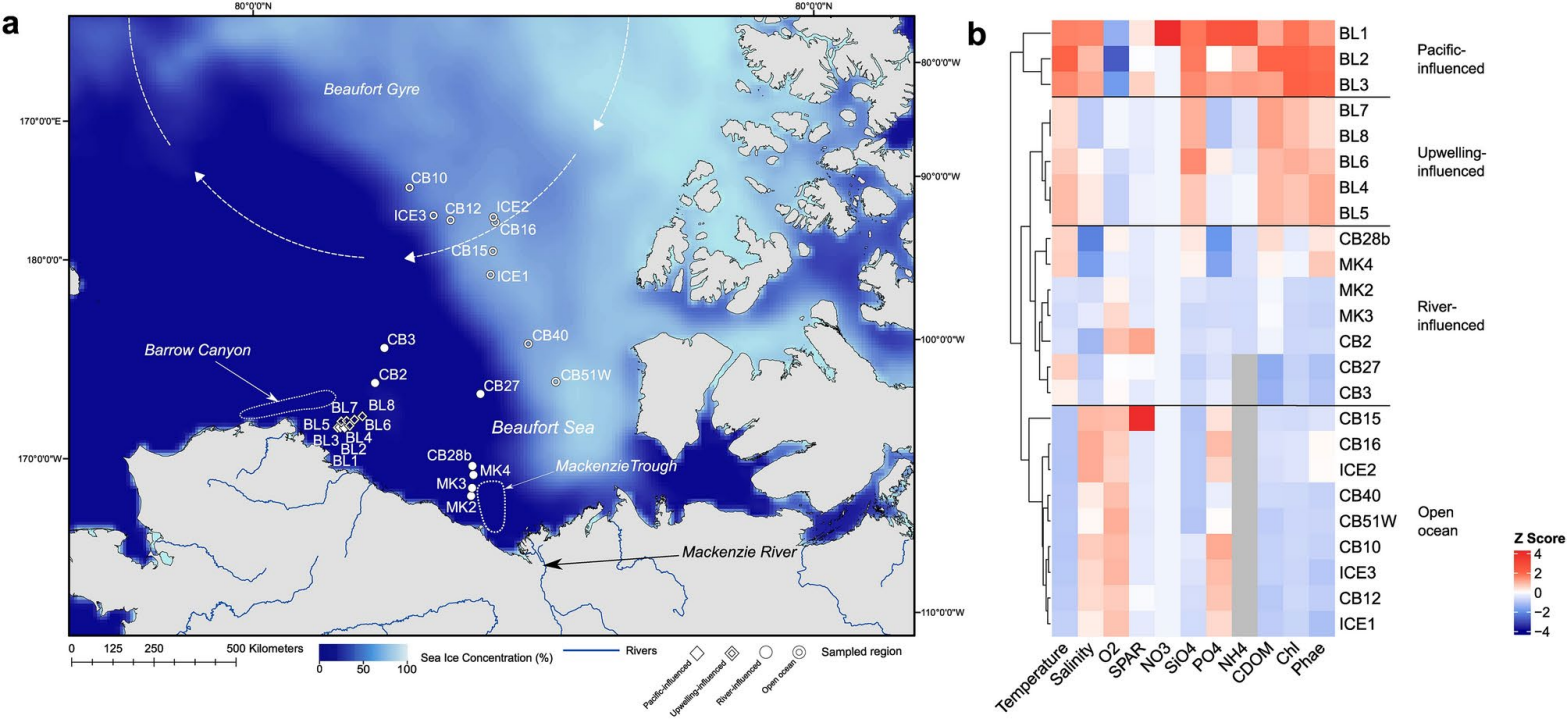
Sampling area and environmental conditions. (**a**) Map of the Beaufort Sea and sampling stations, with average sea ice concentration and notable oceanographic features overlaid. Approximate locations of the Beaufort Gyre, Barrow Canyon and Mackenzie Trough are indicated, and the Mackenzie River is also labelled. Stations are labelled according to the environmental clustering in Fig. 1b. (**b**) Heatmap and dendrogram of standardised environmental data for each station. Cells are coloured according to the z scores calculated for the environmental data matrix (see Table S1). Black dividing lines denote clusters identified by hierarchical clustering of the Euclidean distance matrix. Sampling stations are coloured according to their weighted UniFrac cluster in the bar to the right of the heatmap. Data for NH_4_ were lacking from 11 of the stations, as shown by the greyed-out cells.

Eukaryotic phytoplankton drive AO primary productivity^21,22^, and there is a pressing need to better understand how changing environmental conditions alter the biogeography of microbial eukaryotic communities, in particular at the air-sea interface. However, the phylogenetic composition and structure of surface microbial eukaryotic assemblages occurring across the range of AO ecosystems, including under-sea ice systems, remains understudied. The Beaufort Sea stretches from the coast along northern Alaska (USA) to Nunavut (Canada) and over the Canada Basin to nearly 80° N^23^. Environmental change here has been especially rapid^24,25^, making the Beaufort region a vanguard for understanding change elsewhere in the Arctic^26^. The Beaufort Sea comprises the Mackenzie Canyon, Beaufort Shelf, Amundsen Gulf and the Canada Basin (Fig. 1a)^27,28^. The Beaufort Gyre, one of the major AO features and reservoir of most of the AO’s freshwater^15^, dominates the Canada Basin.

Here, we examine the taxonomic composition and phylogenetic structure of microbial eukaryotic communities recovered from AO surface waters spanning four distinct regions of the 310,000 km^2^ Beaufort Sea. To determine whether the surface AO systems (coastal, river-influenced, open ocean and under-ice) harbour distinct microbial eukaryote communities, we sequenced the V4 region of 18S rRNA from samples collected across the Mackenzie Trough, the Utqiagvik (Barrow) region, the Canada Basin and samples collectively classified as under-ice. Molecular phylogenetics approaches can differentiate between cryptic species or taxa that are difficult to distinguish by microscopy or pigment-based approaches and can also identify the large non-photosynthetic components of the microbial eukaryotic community. Previous molecular taxonomic surveys of microbial assemblages in the Beaufort Sea have detected shifts in community composition associated with environmental perturbations and varied environmental conditions; however, these earlier studies covered small geographic areas^29,30^. Using RNA as a template and targeting 18S rRNA to characterise the metabolically active, living microbial community members in samples^31,32^, we examine microbial eukaryote communities from contrasting regions to determine if specific regions harbour distinct phylogenetic signatures.

## Methods

### Sampling area

Samples were collected in the Beaufort Sea from the icebreaker *CCGS Louis S. St-Laurent* as part of the Joint Ocean Ice Studies (JOIS) program between 12 and 29/09/2018. Open water surface waters (≤5 m depth) were sampled with the ship’s underway pump, while under-ice surface waters were sampled from holes drilled through the ice. 5 L seawater were filtered first through a 50 μm mesh pre-filter, and the material was collected on a 0.4 μm polycarbonate filter. Filters were stored at −80 °C with PowerSoil isolation buffer (Mo Bio Laboratories, Carlsbad, CA USA). Twenty-four stations, including three ice camps on the northern section of the Beaufort Sea, were sampled from four different systems: the Mackenzie Trough (MK, n = 4), the Utqiagvik region (formerly Barrow; BL, n = 8) and the Canada Basin open water (CB, n = 9) and under-ice stations (ICE, n = 3) (Fig. 1a). For the under-ice samples and two BL stations, where ship CTD casts were not done, the environmental data were retrieved from the stations in the immediate vicinity (ICE1: station CB17; ICE2: station CB16; ICE3: station CB9; BL5: station BL4 and BL7: station BL8). Station coordinates and GSHHS shoreline data^33^ were used to map sampling stations in ArcMap. Daily sea ice concentration data during the sampling period were retrieved from the National Snow and Ice Data Center (NSIDC)^34^ and used to calculate the average sea ice concentration across the entire sampling period.

### Environmental data

Ancillary data collected using a conductivity-temperature-depth (CTD) cast from the surface water (≤5 m depth) at each station were obtained from Woods Hole Oceanographic Institution (data are available at www.whoi.edu/site/beaufortgyre/data/ctd-and-geochemistry/; LSSL Geochemistry 2018 dataset). Water samples drawn from Niskin bottles (at the same depth sampled for microbial diversity) were analysed on board for salinity, oxygen and nutrients. Unfiltered nutrient samples (nitrate, silicate and phosphate) were analysed within 12 h of collection onboard using a three-channel nutrient AutoAnalyser 3 (SEAL Analytical GmbH, Norderstedt, Germany) following the methods described by the manufacturer. Ammonium samples were collected along the BL and MK shelf lines: samples of 40.5 (±0.5) mL of seawater were collected in duplicate 50 mL acid-washed Falcon tubes from the Niskin bottles at each station. Ammonium concentrations were measured following the Holmes *et al.* protocol A (0 to 3 µm)^35^, modified for 40 mL sample volume. For chlorophyll and phaeopigments, water was filtered under low pressure onto 0.7 µm pore size GF/F 25 mm filters (Whatman GF/F, GE Healthcare) on board. If water could not be filtered immediately, samples were kept in the dark until filtered within 4 h. Filters were then folded inside a 90 mm grade 1 filter paper (Whatman, GE Healthcare), wrapped in aluminium foil and stored at -80 °C for later analysis onshore. Onshore, the pigment samples were extracted in glass scintillation vials with 90% acetone for 24 h in the dark at -20 °C. Samples were removed from the freezer and placed in the dark one hour before being read using a Field Fluorometer (Turner Designs, San Jose, CA, USA). The environmental metadata (Table S1) were converted to z scores, and the resulting standardised metadata table (Table S2) was used to compute a Euclidean distance matrix with the *vegdist* function in Vegan v2.6–2^36^. Hierarchical clustering was then performed on the distance matrix and used to construct a dendrogram. A heatmap of the standardised metadata table and a dendrogram was constructed using the R ComplexHeatmap package v2.12.1^37^.

### Nucleic acid extraction, amplification and sequencing

Total RNA was extracted from the stored polycarbonate filters with the RNeasy Mini Kit (QIAGEN, Hilden, Germany), DNase treated and reverse transcribed to cDNA using a SuperScript kit and random primers (Invitrogen, Waltham, MA USA). PCR amplification of the V4 region of the 18S rRNA was carried out using the E572F/E1009R V4-18S primers^29^. Library preparation and Illumina MiSeq sequencing were carried out at Dalhousie University’s Integrated Microbiome Resource (Halifax, Nova Scotia, Canada). Sequences are available at the NCBI Sequence Read Archive (SRA) under the accession number PRJNA931954.

### Sequence processing

Sequencing adapters were trimmed using TrimGalore v0.6.5^38^ and primers were specifically removed using Cutadapt v2.10^39^. The reads were then pre-processed using DADA2 v1.14^40^; read quality profiles were visualised before and after filtering and trimming [truncLen = c(280,265), trimLeft = c(18,20), maxEE = c(2,2) and truncQ = 2]. Error rates for forward and reverse reads were then assessed using DADA2. Amplicon sequence variants (ASVs) were identified and forward and reverse reads were merged. Chimeric ASVs were removed, and the PR^2^ reference database v4.12.0^41^ was used for taxonomic assignments with the RDP classifier^42^ (output available at 10.5281/zenodo.7612768). ASVs classified as multicellular taxa (Metazoa, Streptophyta) were discarded from this study.

An operational taxonomic unit (OTU) approach was also used to verify the results of the ASV-based approach. Briefly, the sequences from the DADA2 sequence table were clustered at 98% similarity (*TreeLine* function) following alignment (*AlignSeqs* function) and distance matrix generation (*DistanceMatrix* function) using the DECIPHER v2.24.0 R package^43^. The resulting OTU table was then filtered to remove Metazoa and Streptophyta.

### Phylogenetic community analyses

ASVs were aligned to the 18S Silva reference alignment (Release 102)^44^ using MAFFT v7^45^ with the G-INS-i iterative refinement method. The ASV maximum-likelihood phylogenetic tree was reconstructed using IQ-TREE v2.0^46^ with 1000 non-parametric bootstrap replicates and using the TIM2 + F + R10 model, which was evaluated as the best-fit model by ModelFinder^47^ and BIC criterion. Bray–Curtis, unweighted and weighted UniFrac distance matrices were computed using the ASV table with Phyloseq v1.40.0^48^. The homogeneity of variance (homoscedasticity) between the four communities was assessed using the *betadisper* function in Vegan and an ANOVA test. A PERMANOVA test (Vegan R package; *adonis* function) was used to assess if the community composition differed significantly between Beaufort Sea habitats. The distance matrices were then ordinated and plotted as a non-metric multidimensional scaling (NMDS) plot using the *ordinate* and *plot_ordination* functions in Phyloseq. The R *hclust* function was used for the hierarchical clustering of the distance matrices. Additionally, samples were clustered using the UPGMA method in QIIME 2 2023.9^49^ (*q2-diversity* plugin) at a rarefaction depth of 20,000 sequences with 1000 iterations. The OTU table was also used to compute a weighted UniFrac distance matrix, which was ordinated and plotted as an NMDS using Phyloseq v1.40.0. Samples were clustered using the UPGMA method in QIIME 2 2023.9^49^ (*q2*-*diversity* plugin) at a rarefaction depth of 20,000 sequences with 1000 iterations.

Species richness measures were calculated on the ASV table with samples rarefied to an even depth of 25,000 sequences. The mean number of ASVs (species richness; SR_25k_), mean Faith’s phylogenetic diversity (PD_25k_) and mean Simpson index were calculated from 100 rarefaction iterations over each sample, using the *rarefy_even_depth* and *estimate_richness* functions from Phyloseq, and the Picante R package v1.8.2^50^ function for PD. We used Picante to calculate the standardised effect sizes of phylogenetic diversity (*SES*_*PD*_) on rarefied and non-rarefied ASV tables to identify if rarefaction affected the alpha diversity estimates. Standardised effect size of mean pairwise distances (*SES*_*MPD*_)^51^ were calculated per cluster with 1000 iterations using the non-rarefied ASV table (converted to relative abundances). Relative abundances per sample were randomised 1000 times and used to calculate the mean NRI (-1 × *SES*_*MPD*_)^52^ of each community cluster. NRI is a measure of phylogenetic relatedness of species within a community^52^; it reflects the degree to which species (or here, ASVs) in a community are more closely related to each other than would be expected by chance. Positive NRI values indicate phylogenetic clustering and suggest that abiotic environmental filtering is a dominant process in shaping community composition^53^. Negative NRI values indicate phylogenetic overdispersion where either competitive exclusion acting on evolutionarily conserved traits, or environmental filtering acting on evolutionarily convergent traits plays an important role^53^.

### Non-negative matrix factorisation

We used a non-negative matrix factorisation (NMF) approach to partition the samples using the NMF R package^54^. NMF is a dimension reduction method that decomposes the ASV abundance matrix in the product of 2 matrices. The coefficient matrix describes the overall structure of the abundance matrix with a small number of descriptors (the number of descriptors is the rank of the NMF decomposition). The basis matrix gives the weights of each original descriptor (ASV) on the new descriptors. NMF has been used to describe the spatial structure of microbial communities’ compositions (microbial species as descriptors^55^). NMF analysis was first computed with rank values ranging from 3 to 7, 100 runs, and various algorithms (nsNMF, Brunet, KL). The optimal results were obtained for the nsNMF algorithm, random seed of the factorised matrices, and a rank value of 4. We performed the final analysis with 200 runs, a rank of 4, random seed and the nsNMF algorithm.

The ASV indices were calculated by combining two methods described in Jiang et al*.*^56^ and Kim et al.^57^: (1) the Spearman correlation coefficient between an ASV (*i*) and a descriptor (*k*) ρ_*i,k*_ was calculated using the abundance profile of the ASV (original abundance matrix) and SC (coefficients matrix) along all the samples; (2) The multidimensional projection between an ASV and a descriptor was calculated as the cosine of the angle between the vectors represented by an ASV abundance in the sample space and the vector represented by descriptors in the sample space. The abundance profiles of ASVs and descriptors were first normalized, and we calculated the multidimensional projection as:

$${Cos\theta }_{i,k}=\sum_{j=1}^{n}{a}_{i,j}\times {s}_{k,j}$$

where *Cosθ*_*i,k*_ is the multidimensional projection between the ASV *i* and the descriptor *k, n* is the number of samples,* a*_*i,j*_ is the normalized abundance of the ASV *i* in the sample *j,* and* s*_*k,j*_ is the normalized abundance of the descriptor *k* in the sample *j.* We then used the basis matrix to calculate the score of each ASV:

$$Score\left(i\right)=1+\frac{1}{{\text{log}}_{2}(q)}\sum_{k=1}^{q}p\left(i,k\right){\text{log}}_{2}(p\left(i,k\right))$$

where *i* is the ASV, *q* is the number of descriptors (4 in our study), *k* is the descriptor number, *p(i,k)* is the probability of finding the ASV *i* in the descriptor *k*. The final index of ASV *i* on descriptor *k* (*I*_*j,k*_) was obtained by multiplying the Spearman correlation coefficient, cos theta and the ASV score:

$${I}_{i,k}={\rho }_{i,k}\times {Cos\theta }_{i,k}\times Score\left(i\right).$$

To select the ASVs with the greatest index values for each descriptor, the distributions of ASV indices for each descriptor were plotted (Fig. S1) and means and standard deviations calculated. The ASVs with index values greater than three standard deviations above the mean of each descriptor were classified as signature ASVs for their corresponding descriptor.

### Functional structure, rare taxa and spatial pattern analyses

In addition to taxonomic and phylogenetic analyses, the data were examined from a functional perspective. Using an 18S functional association database^58^ to obtain information on chloroplast presence/absence for each sequence identification number in the PR^2^ database, ASVs were assigned as either ‘chloroplast-containing’ (including mixotrophs) or ‘heterotroph’ taxa. We carried out beta-diversity analyses and calculated NRI for both subsets of ASVs.

We conducted community analysis on rare ASVs, defined as those with a total abundance of less than 0.01% across all samples^59^. We used UniFrac (weighted and unweighted) and Bray–Curtis distance methods to analyse the beta-diversity patterns of rare taxa within the dataset. We also calculated mean NRI values for the rare biosphere.

A distance matrix of geographic distances between sample locations was calculated in R using the *geodist* function from the geodist R package^60^. Geographic distances between samples were then plotted against community dissimilarities (weighted UniFrac distances) and the *decay.model* function in the betapart R package^61^ was used to fit a model of distance-decay to the data. A multiple regression on distance matrices (MRM) was carried out with the ecodist R package^62^ to assess the strength of the relationship between geographical distance and community dissimilarity.

## Results

### Environmental context

Samples were collected from surface waters at 24 stations (Fig. 1a), representative of four habitats found across the Beaufort Sea: 1) open ocean Canada Basin (CB); 2) adjacent under-ice communities in the Canada Basin (ICE); 3) and 4) and two separate onshore to offshore river-influenced sites. Although the ICE stations were geographically close to several of the CB stations, the samples were taken from the water directly underneath the surface of the sea ice, whilst all other samples were retrieved from the ice-free surface water at the top of the polar mixed layer using the ship’s underway pump. The Mackenzie (MK) samples were coastal and within an area influenced by the Mackenzie River plume, adjacent to the Mackenzie Trough. The Barrow (BL) stations, situated off the coast of Nuvuk (formerly Point Barrow, Alaska, USA), were also coastal and near the Barrow Canyon. The BL stations were the most westerly stations of this study and the most influenced by Pacific Waters from the Bering Strait. Both canyon and trough systems are influenced one after the other by the east-flowing Beaufort shelf break jet as well as asymmetric upwelling of the canyons^63^. Average sea ice concentrations across the Beaufort Sea varied, with much greater concentrations of ice present in the eastern regions and more open water to the west (Fig. 1a).

The standardised environmental data heatmap (Fig. 1b) separated the most coastal, Pacific-influenced sites within the BL region (stations BL1, BL2 and BL3). These stations had 14% more silicate, 13% more phosphate and 95% more ammonium than the other BL stations. BL1 had the highest nitrate levels (0.0986 mmol/m^3^) of all the stations in this study. BL1, BL2 and BL3 also had higher mean chlorophyll *a* (mean *µ* = 1.12 µg/L; standard deviation σ = 0.06) and phaeopigment (*µ* = 0.35 µg/L; σ = 0.04) concentrations when compared to all other stations, where chlorophyll *a* was measured at ≤0.6 µg/L and phaeopigment at ≤0.29 µg/L. The other BL stations were further offshore and closer to the Barrow Canyon upwelling, and were significantly colder (*µ* = 0.3 °C, σ = 0.2) than the inshore BL stations (*µ* = 1.4 °C, σ = 0.3; t(3.6) = -6.33, *p* = 0.004). The upwelling-influenced stations (BL4-8) were also significantly fresher (*µ* = 26.97 PSU, σ = 0.7) than the Pacific-influenced stations (BL1-3; *µ* = 28.48 PSU, σ = 0.4; t(6.0) = -4.18, *p* = 0.006). Samples from the Mackenzie Trough region clustered together with stations CB2, CB3 and CB27 from the open water CB region, forming the river-influenced cluster. These CB stations were closer to the shelf break and were fresher (*µ* = 26.17 PSU, σ = 0.3) than the other CB stations (*µ* = 27.83 PSU, σ = 0.4; t(5.2) = − 6.68, *p* = 0.001). Stations CB28b and MK4 were fresher (*µ* = 25.07 PSU, σ = 0.3) than the other river-influenced stations (*µ* = 26.37 PSU, σ = 0.8; t(2.2) = -4.62, *p* = 0.036). CB28b and MK4 also had higher temperatures (*µ* = 0.22 °C, σ = 0.05) than the other river-influenced stations (*µ* = -0.59 °C, σ = 0.58; t(4.1) = 3.10, *p* = 0.03), as well as 28% higher silicate, 30% higher CDOM and 78% higher phaeopigment concentrations. The northernmost CB stations (CB10, CB12, CB15 and CB16) had the highest phosphate concentrations outside of the BL system (*µ* = 0.55 mmol/m^3^, σ = 0.01). The CB and ICE stations in the Canada Basin region formed an open ocean cluster characterised by 5% greater O_2_ levels than the BL stations and the lowest mean temperature (*µ* = -1.5 °C, σ = 0.07) of all the clusters (river-influenced *µ* = -0.3 °C, σ = 0.62; upwelling-influenced *µ* = 0.3 °C, σ = 0.21; Pacific-influenced *µ* = 1.4 °C, σ = 0.25).

### Phylogenetic biogeography and diversity

Taxonomic assignment and filtering of 18S rRNA ASVs resulted in a final sequence table with 1449 microbial eukaryote ASVs from ~1.4 million reads. Approximately 21% of ASVs could not have their taxonomy fully assigned (to species level). Variance within the four communities was homogeneous (*betadisper* and ANOVA; F = 0.56, *p* = 0.65), but the communities differed significantly in composition (PERMANOVA; R^2^ = 0.713, F = 16.57, *p* < 0.001).

**Fig. 2:**
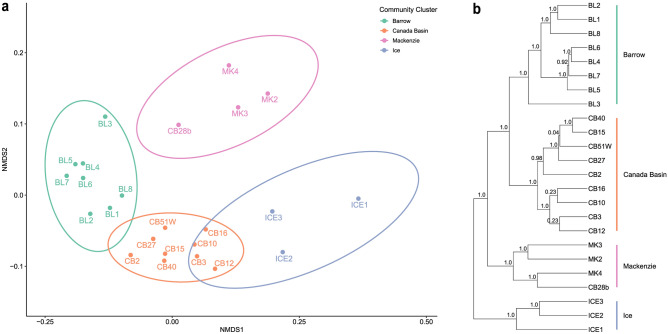
Phylogenetic beta diversity based on ASV analyses. (**a**) NMDS ordination of the weighted UniFrac distance matrix (stress = 0.086). Samples are coloured by their weighted UniFrac cluster and ellipses represent 95% confidence intervals around each cluster. (**b**) Consensus tree of UPGMA sample clustering using weighted UniFrac as the metric and a rarefaction depth of 20K with 1000 iterations. Branch support values are indicated.

We assessed phylogenetic beta diversity using weighted UniFrac distances. NMDS ordination and UPGMA clustering were used to identify four distinct microbial communities (Fig. 2). The microbial assemblages collected from open-ocean waters grouped together, forming a distinct CB cluster, while samples taken from waters directly beneath the sea ice grouped apart, forming the ICE cluster. Samples from the MK transect grouped together, as did the BL samples. The OTU-based sample clustering yielded near identical results to the ASV approach in the weighted UniFrac NMDS and UPGMA sample clustering (Fig. S2). There was a positive trend between weighted UniFrac and geographic distances of the samples, as seen on the distance-decay plot (Fig. S3; *p* < 0.001, slope = 2.99 × 10^–4^). Furthermore, a multiple regression on matrices (MRM) carried out on the phylogenetic and geographic distance matrices also showed a significant relationship between the two (MRM; R^2^ = 0.107, F = 97.88, *p* < 0.05).

We analysed the community partitioning using unweighted UniFrac and Bray–Curtis distances (Fig. S4). Except for the BL samples, which clustered together, the samples did not form distinct clusters in the NMDS plot of unweighted UniFrac distances (Fig. S4a). Instead, the MK stations clustered with CB and ICE stations. In the Bray–Curtis NMDS plot (Fig. S4b), the sample clusters were more clearly defined, with the MK stations forming a cluster and most BL stations clustering together.

**Fig. 3:**
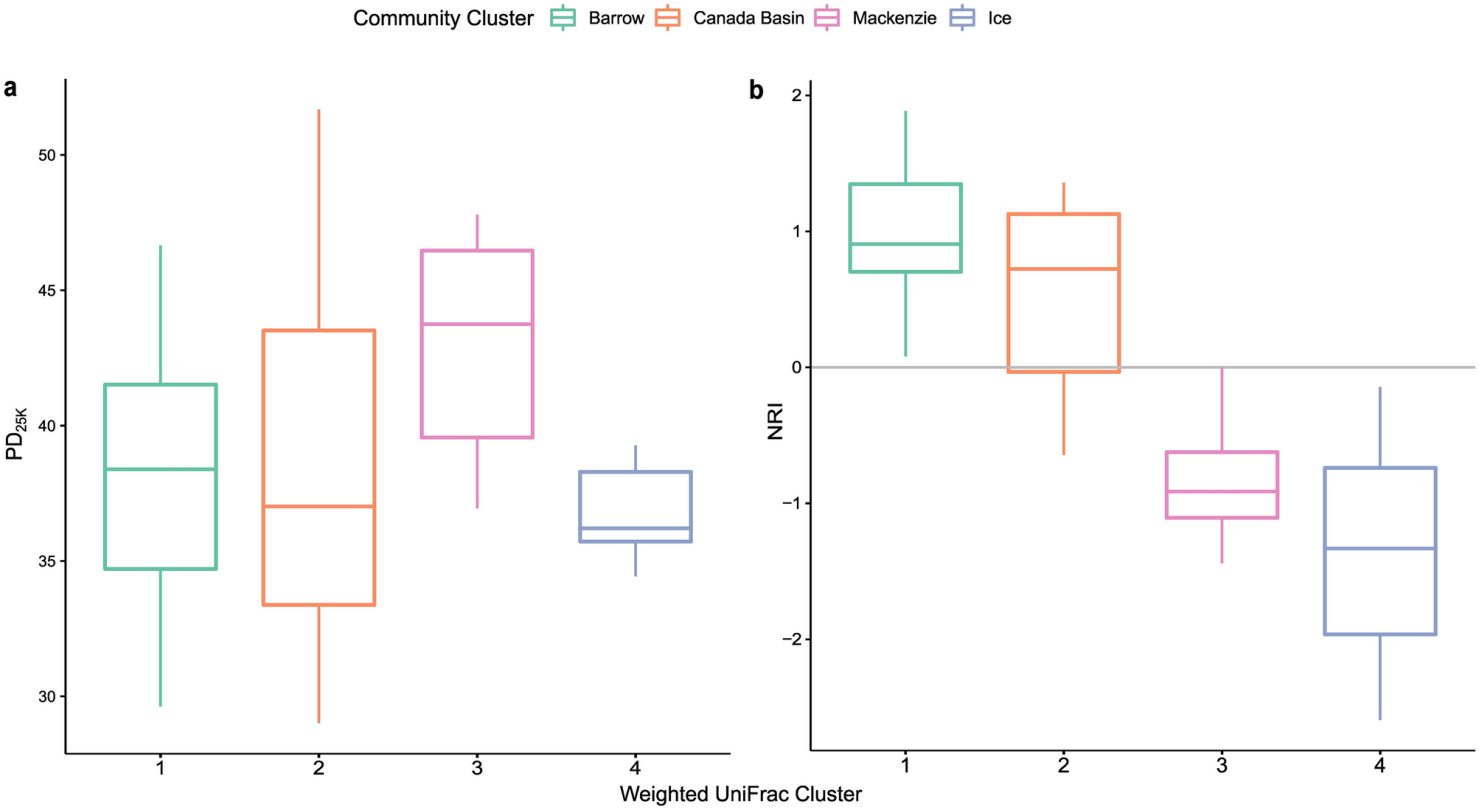
Boxplots of alpha diversity measures of the community clusters. (**a**) Phylogenetic diversity of samples grouped by community cluster and rarefied to 25,000 sequences. (**b**) Net relatedness index (NRI) of samples grouped by community cluster.

To determine which community cluster (Fig. 2) was the most phylogenetically diverse, we calculated Faith’s phylogenetic diversity (PD_25k_; Fig. 3a). Mean PD_25k_ varied significantly between clusters (Fig. 3a; ANOVA, *p* < 0.0001, F = 129). The MK community had the highest phylogenetic diversity compared with the other three communities (Tukey test,* p* < 0.0001; MK PD_25k_ = 43.1, σ = 3.5; BL PD_25k_ = 38.3, σ = 5.0; CB PD_25k_ = 38.1, σ = 6.0; ICE PD_25k_ = 36.7, σ = 1.3) and the highest mean species richness (SR_25k_ = 333.5, σ = 54.6; Fig. S4a). The lowest SR_25k_ was seen in the ICE community (SR_25k_ = 283.3, σ = 9.1). The greatest range in PD_25k_ (29.0—51.7) was observed in the CB cluster, but there was no significant difference between CB and BL clusters. *SES*_*PD*_ values for each cluster were comparable between the rarefied and non-rarefied ASV tables (Fig. S6). We also calculated the net relatedness index (NRI) for each of the four clusters of samples in the weighted UniFrac plot (Fig. 3b). Mean NRI varied significantly between clusters (Fig. 3a; ANOVA, *p* < 0.001, F = 9.86). Positive NRI values, as observed for BL and CB clusters, suggest that abiotic environmental filtering was important in shaping community composition^53^, whereas negative NRI values observed in ICE and MK clusters suggest that either competitive exclusion acting on evolutionarily conserved traits, or environmental filtering acting on evolutionarily convergent traits was important in determining community composition^53^. There were significant differences in mean NRI between the BL and MK clusters (Tukey test, *p* < 0.01) and between the CB and MK clusters (Tukey test, *p* < 0.05). The ICE cluster also had significantly lower NRI values than those from BL and CB clusters (Tukey test, *p* < 0.01 and *p* < 0.001, respectively).

### Functional and rare biosphere community analyses

**Fig. 4:**
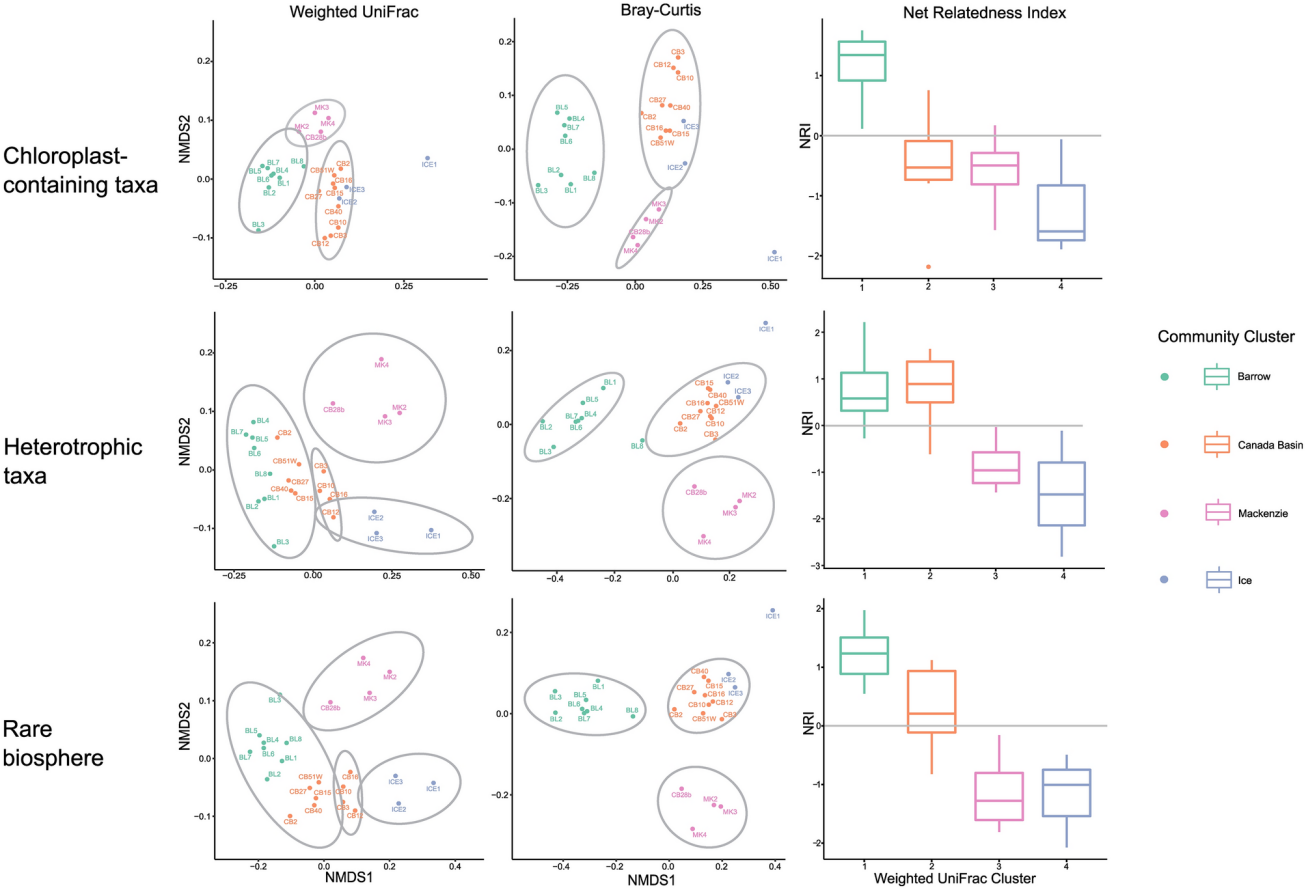
Beta diversity and net relatedness index (NRI) of trophic subsets of taxa and the rare biosphere. For each of the three subsets (chloroplast-containing taxa, heterotrophs and rare taxa), a weighted UniFrac and Bray–Curtis NMDS ordination, and a boxplot of NRI values is shown.

To determine if proximate trophic modes influenced community composition, each ASV was labelled as either chloroplast-containing (inclusive of mixotrophs) (n = 227) or heterotrophic (n = 1222), based on its taxonomic classification. Additional phylogenetic analyses were carried out on these two categories. When considering only chloroplast-containing taxa, ICE1 clustered apart from all other samples in the weighted UniFrac and Bray–Curtis NMDS ordinations (Fig. 4). In ordinations, the BL and MK samples also formed distinct clusters, whereas the CB samples formed clusters with ICE2 and ICE3 samples. All community clusters (except BL) had negative mean NRI values when examining exclusively chloroplast-containing taxa. The heterotrophic taxa from MK samples clustered together in both weighted UniFrac and Bray–Curtis NMDS ordinations. All three ICE samples clustered together in the heterotroph-only weighted UniFrac NMDS, albeit with some overlap with some CB samples. Both ICE1 and BL8 were distinct from their respective clusters in the Bray–Curtis NMDS. The ICE and MK heterotrophic communities had negative mean NRI values in contrast with the BL and CB communities, the latter of which had the highest mean NRI of the heterotroph-only communities.

To test the hypothesis that rare taxa significantly influenced phylogenetic community structure, we carried out the same phylogenetic analyses on the rare biosphere, defined here as the ASVs with <0.01% relative abundance across all samples (n = 1203)^59^. In the NMDS ordination of the weighted UniFrac distances, the MK and ICE clusters formed distinct and exclusive clusters. The CB samples were split across two clusters, however. Four CB samples clustered together in their own group, whereas the other five clustered with the BL samples. In the Bray–Curtis NMDS ordination, the BL and MK samples formed distinct clusters, and the CB samples clustered with ICE2 and ICE3, leaving ICE1 separate from the other clusters. The BL community had the highest mean NRI and both MK and ICE communities had negative mean NRI values. The mean NRI for the CB community was above zero but the range of values among samples went from negative to positive.

### Signature sequence variants

**Fig. 5:**
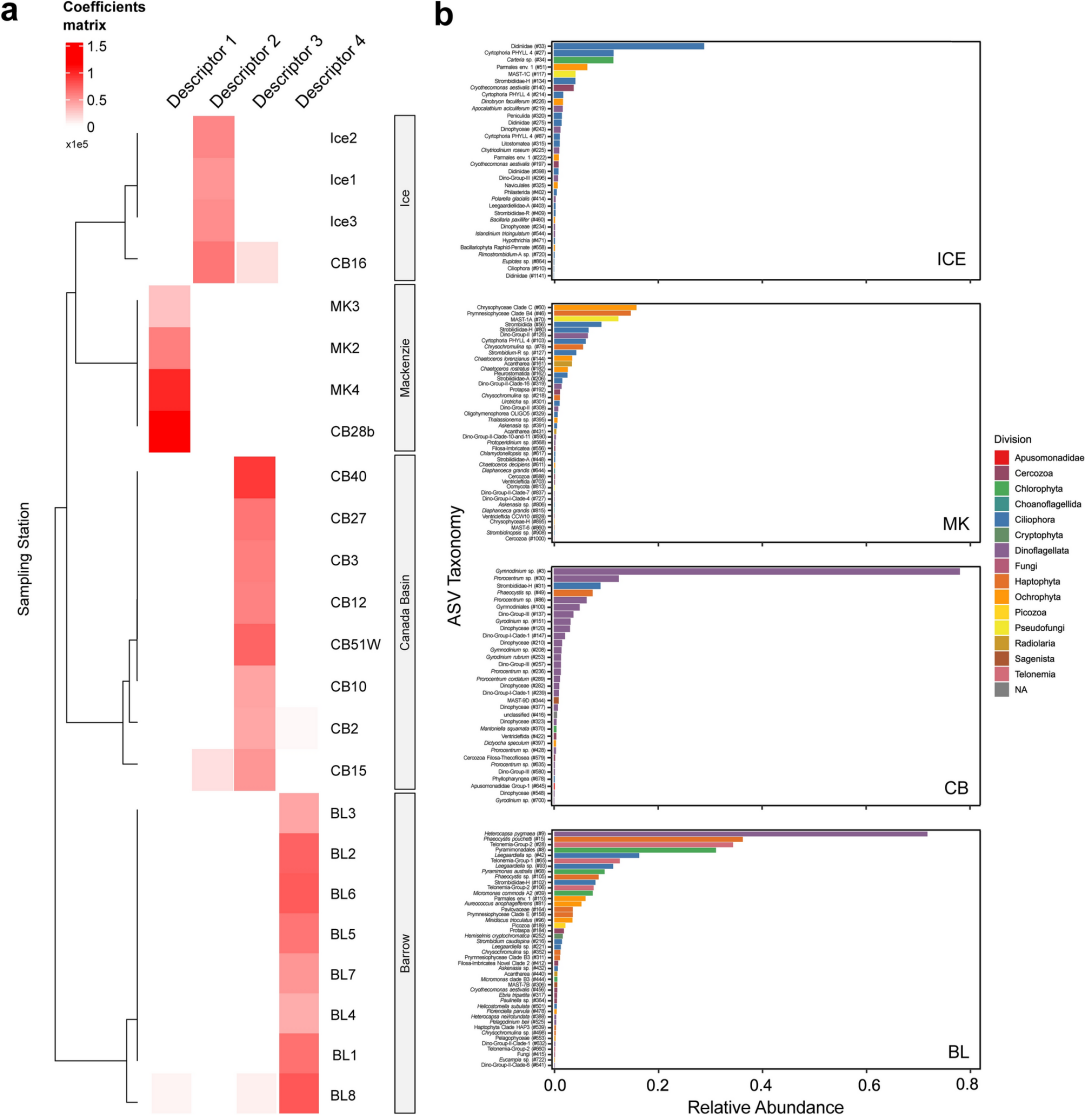
NMF analysis and signature ASVs. (**a**) Heatmap of the NMF coefficients matrix with cells coloured according to the correlation coefficients of each sampling station to each descriptor. (**b**) Bar plots showing the relative abundances of signature ASVs in each descriptor as determined by the NMF analysis. Bars are coloured according to the phylogenetic division of the ASV determined using the PR2 database. The ASV numbers of each signature ASV are indicated in parentheses.

**Fig. 6:**
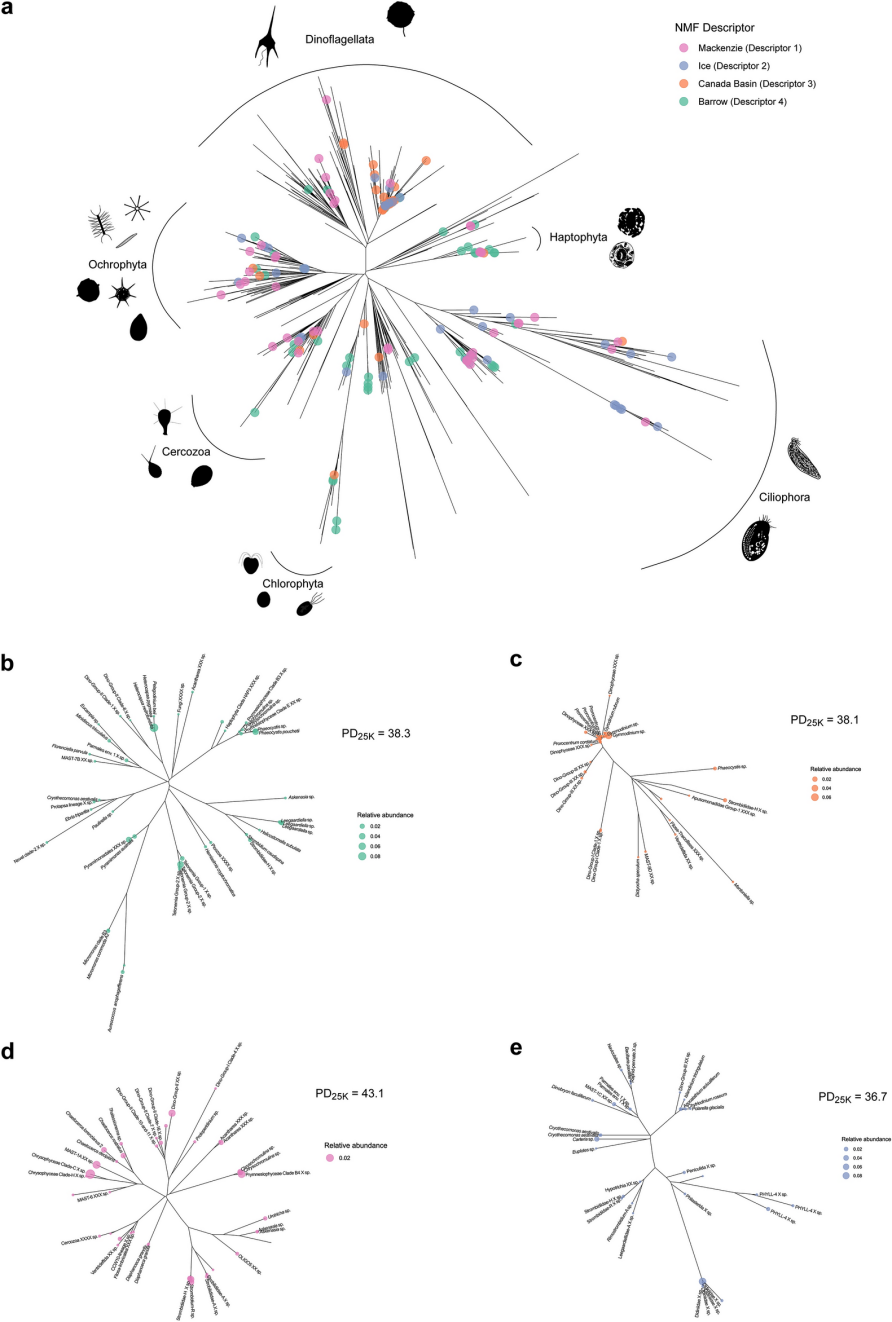
Maximum-likelihood phylogenetic reconstructions of ASVs. (**a**) Phylogenetic tree of all ASVs identified across all samples in this study, with signature ASVs for each NMF descriptor denoted by coloured circles on the leaves. Major clades are also indicated on the tree with images of representative species for each clade shown. (**b**–**e**) Sub-trees of Fig. 6a showing signature ASVs in each descriptor. Leaf size is proportional to the ASV’s relative abundance in the descriptor, and PD_25K_ of the whole descriptor is also shown.

We then applied a non-negative matrix factorisation (NMF) analysis to identify key taxa (signature sequence variants based on Fig. S1) in each community cluster (Figs. 5 and 6). This method groups samples based on the contribution of descriptors in each sample, and we found that there was a strong agreement between the clusters identified using weighted UniFrac and the descriptors from the NMF analysis, with the only exception being that station CB16 aligned more closely with the ICE community than with the CB community, but also retained affinity to the CB community.

The BL community had the most signature ASVs (n = 44), of which 17 originated from photosynthetic taxa (Fig. 6b, Table S3). *Micromonas* clade B3 had the third greatest ASV index value (ASV_444; index = 0.78) and was unique to the BL signature community. The majority of the 33 signature ASVs in the CB community were from dinoflagellates, including species of the genera *Gymnodinium* and *Prorocentrum*, with many other unclassified dinoflagellate ASVs (Fig. 6c, Table S4). The taxon with the greatest ASV index value for the CB community was *Mantoniella*, a chlorophyte alga belonging to the Mamiellophyceae^64^. Many of the 42 MK signature ASVs were heterotrophic species such as ciliates and choanoflagellates, as well as nominally parasitic dinoflagellates in the Syndiniales (Fig. 6d, Table S5). There were four diatoms among the MK signature ASVs, three of which were *Chaetoceros* spp., as well as three prymnesiophytes. Among the 34 ICE community signature ASVs, the only chlorophyte was classified as *Carteria* sp. (Chlamydomonadales). The signature diatoms of the ICE community were an unclassified pennate diatom, *Naviculales* sp. and *Bacillaria paxillifer*. Most of the signature ASVs in the ICE community originated from heterotrophic or mixotrophic taxa, almost all dinoflagellates and ciliates (Fig. 6e, Table S6).

## Discussion

Our results highlight the critical link between biodiversity and ecosystem resilience, suggesting that areas with less diverse assemblages, such as those under ice, are at greater risk of disruption due to environmental perturbations. By leveraging amplicon sequence data, we inferred species or ecotype presence, absence, and relative abundance, which allowed us to determine patterns of biodiversity and biogeography across the surface Arctic Ocean. Using phylogenetic approaches, we identified subtle diversity differences across regions, effectively distinguishing between under-ice stations and open-water microbial communities. Our analysis suggests that microbial communities from under ice ecosystems may be particularly susceptible to replacement under borealisation of the AO^65,66^, underscoring the vulnerability of these ecosystems in the face of rapid environmental change.

### Challenges in microbial community studies

Using amplicon sequence data has proven to be a fundamental tool for understanding ecological dynamics across various environments^67–69^, and is of particular importance in under sampled polar regions to provide initial perspectives on community diversity, which may subsequently be validated by qPCR/dPCR^70^. Although the use of rRNA as a marker gene could skew diversity estimates due to copy number variation between taxa, the effect of multiple rRNA gene copies may in fact contribute little to the combined bias incurred from each methodological step in metabarcoding studies^71^. Since rRNA gene copy number tends to increase with phytoplankton cell size, we focused on cells <50 µm, which in theory harbour fewer rRNA gene copies than larger cells^72,73^.

In this study, we primarily use ASVs over OTUs. Although OTUs may reduce the likelihood of discovering false biological diversity by combining similar amplicon sequences into taxonomic units, they may simultaneously overlook the fine-scale variation that exists between microbial strains, which can be distinguished with modern high-quality sequencing methods^40,74^. Intragenomic rRNA polymorphisms may also inflate the diversity detected in a dataset if different gene copies are classified as separate ASVs/OTUs. However, using even a strict cut-off of genetic distance of 0.01 to cluster OTUs should not create artificial diversity because the mean genetic distance between multiple 18S rRNA copies in the same genome is less than 0.003, meaning that multiple copies from the same genome should cluster as a single OTU^75^. We used rRNA instead of rDNA metabarcoding to examine the diversity of predominantly living and metabolically active community members^32,76,77^. Studies using both rRNA and rDNA have found both general agreement and differences between the two methods^77–80^; some of the observed differences may have been overlooked if only rDNA had been studied^76^. Moreover, rRNA sequencing has been suggested to be superior to rDNA approaches^81^, for instance, in its sensitivity to environmental conditions^82,83^. This is an important factor to note in the context of a changing AO. Errors in RNA transcription could cause overestimations of biodiversity as these would likely not be detected and corrected by the DADA2 algorithm. However, transcriptional errors remain uncommon. In eukaryotes, most rDNA genes are transcribed by RNA polymerase I; a study on transcription errors in yeast found the error rate of RNA polymerase I to be 4.3 × 10^–6^ per base pair^84^.

Despite these challenges, our results remain robust when using OTUs clustered at 98% similarity, with the same overall patterns of diversity observed. Future microbial community studies of the AO may opt to utilise rDNA and rRNA in parallel to minimise the impact of sequence artefacts from dead cells while also allowing researchers to compare ‘total’ and ‘active’ microbial communities in contrasting environments and seasons^76,80,85^. There is, however, still a need for standardised methods in microbial ecology, from field sampling to laboratory and bioinformatic protocols^86^.

### Contrasting marine systems

Previous studies in the Canada Basin area focused on the vertical (different depths in the water column) diversity of microbial eukaryotic communities that vary depending on the water mass of origin and are relatively easy to identify in this region of the Arctic^30,87^. In contrast, our study focused on microbial eukaryotic communities’ horizontal diversity across the Beaufort Sea. We identified three geographic regions using abiotic factors: (1) Canada Basin; (2) Mackenzie trough and Beaufort shelf stations; and (3) the coastal Barrow Canyon. The surface microbial communities only partially followed these divisions and could be separated as Barrow Canyon, Mackenzie-influenced, open-water and under-ice Canada Basin microbial communities. These results highlight the variability of microbial communities across the surface AO, even within the short 18-day period in which these samples were taken. Whilst our snapshot precludes predicting how these communities may adjust to ongoing ocean surface warming and freshening over an annual cycle, the study indicates community complexity and diversity associated with areas of the Beaufort Sea. The proportion of sequences (~21%) in our dataset whose taxonomy could not be assigned to species level by the reference 18S database has been noted previously in the extensive pan-Arctic sampling effort by Tara Oceans^88^, and suggests a high proportion of AO eukaryotes remains to be characterised. This highlights the need for ongoing culture and sequence validation as recently reported for deep branching predatory flagellates in the world ocean, including in the Arctic^89^. Culture-independent methods such as single-cell omics also play a vital role in characterising the extent of microbial eukaryote diversity, since these methods are not reliant on available reference genomes or cultured isolates^90–93^.

High Chl *a* and phaeopigments indicated an area of potential high productivity and potential phytoplankton turnover in coastal waters from the Alaskan Beaufort Sea (stations BL1, BL2 and BL3)^94^. The relatively higher nutrient concentrations in the coastal BL stations indicate an influence of nutrient-rich Pacific water flowing into the Beaufort Sea through the Bering Strait and Chukchi Sea^95,96^. However, our sampling was somewhat late in the season, and the high Chl *a* concentrations at the surface suggest ongoing nutrient input and local surface warming in the stratified water column. The Alaskan Beaufort Sea, where the BL samples were taken, experiences periodic upwellings driven by winds, particularly in the late autumn^97,98^. These upwelling events bring denser and more nitrate-rich Atlantic water to the surface of the Nuvuk region^99^. As surface nutrients usually are depleted during the productive spring and summer months, such upwelling events may locally replenish nutrients to the surface waters of the Nuvuk region in the late summer, allowing primary productivity to continue later than in other regions. This is consistent with our data, where saltier, nutrient-rich waters were seen at the coastal BL stations compared to other stations. The relatively fresher, cooler and more oxygenated waters of the offshore BL stations (BL4-BL8) suggest a region of upwelling close to the Barrow Canyon. The BL microbial community had the greatest number of photosynthetic signature ASVs, which, along with the greater concentrations of Chl *a* and phaeopigments, indicates an area of higher primary productivity and grazing and degradation^100^. The BL community also had more signature ASVs than any other communities, reflecting a high level of uniqueness of the Barrow taxa to these samples.

Despite the BL and MK communities both being coastal systems influenced by rivers and upwelling, they were distinct from each other. Although higher temperatures were also observed at MK4 and CB28b (the Mackenzie River offshore stations), lower salinity levels accompanied by high CDOM concentrations were consistent with an offshore upwelling of warmer, fresh water from the Mackenzie River plume, as previously suggested^101^.

The MK community had the second highest number of signature ASVs, mostly from heterotrophic taxa, in contrast with the BL community, which was dominated by chloroplastidic groups. In addition, the MK community had higher mean PD_25k_ and SR_25k_ values than the BL community but had a negative NRI, indicative of phylogenetic overdispersion (evenness)^52,102,103^. This may help explain why this community was more diverse than any others. Phylogenetic overdispersion commonly occurs when competition regulates community membership, and ecologically similar taxa cannot coexist, leading to a phylogeny where species are more distantly related than would be expected by chance (competitive exclusion)^53,104^. However, overdispersion may also result from distantly related species having convergent traits allowing them to survive in their environment, in which case habitat filtering may be driving MK community structure^103,105^. Another explanation could be that input of freshwater species from the Mackenzie River and the development of a brackish water community^16^ led to phylogenetic overdispersion, as previously noted in Mackenzie Shelf bacterial communities^85^.

By the same measure, the positive NRI of the BL community indicated phylogenetic clustering, where species are more closely related than expected by chance; given the distinctive environmental conditions measured in the BL samples, habitat filtering was the more likely driver of community structure here. The BL community was also the only community that was distinct from the others in all three beta-diversity measures used, implying that its structure differed both qualitatively and quantitatively from the other communities^106^. This may have been due to the influences of Pacific water and localised upwelling, which provided an influx of nutrients that sustained a community characterised by phytoplankton.

### Functional perspectives on community structure

To relate phylogenetic community structure to ecological function, we carried out beta-diversity analyses on subsets of taxa based on their proximate trophic status. Chloroplast-containing (including mixotroph) taxa played essential roles in structuring the BL and MK communities, as these samples formed distinctive clusters in both weighted UniFrac and Bray–Curtis NMDS plots (Fig. 4). ICE1 clustered apart from the other ICE samples in the chloroplast-only plots, indicating that its community composition was both qualitatively and quantitatively distinct from the communities recovered from other ice-covered surface waters. *Carteria* sp., a chlorophyte alga, appeared only in ICE1 and is therefore likely to have contributed to differentiating this sample from others. *Carteria* has previously been found to be abundant in Arctic summer sea ice melt ponds, which can become connected to seawater and contribute algae to the under-ice community^107^. A possible explanation for the presence of *Carteria* in only one of the ICE communities is that the under-ice habitat here may have been receiving meltwater from the sea ice surface containing *Carteria*, suggesting connectivity between upper and lower sea ice surfaces^108^. Another difference in chloroplast-containing taxa between ICE1 and ICE2/ICE3 was the presence of haptophyte species such as *Phaeocystis* spp. and *Chrysochromulina* spp. in ICE2/ICE3, neither of which were detected in ICE1. *Sarcinochrysis* sp. and two other unclassified pelagophytes were also found in ICE1 but not in ICE2 or ICE3. One of the unclassified pelagophytes in ICE1 (ASV_1365) was subsequently found to have >99% sequence identity with the Arctic pelagophyte *Plocamiomonas psychrophila* (CCMP2097)^109^, which is a euryhaline species likely adapted to living in ice^110^. These findings are indicative of a unique assemblage in the ICE1 sample. Arctic genotypes of *Phaeocystis* and *Chrysochromulina* have been previously recorded^111^, and *P. pouchetii* dominated phytoplankton communities during the 2007 record ice melt^112^. Moreover, *P. pouchetii* abundances rapidly increased during sea ice retreat in the Barents Sea^113^, suggesting that this taxon may be associated with sea ice melt.

The predominance of heterotrophic and parasitic signature ASVs in the MK and ICE communities (Fig. 6d,e) may result from environmental factors limiting phytoplankton growth, such as decreased light levels and nutrient availability. In coastal MK and under-ice ICE stations, the surface waters had low photosynthetically active radiation (PAR) measures. The sea-ice cover limited the light intensity for the ICE stations, whereas the turbidity introduced by the Mackenzie River plume likely limited light levels. This reduction in light constrains the growth of photosynthetic organisms, resulting in an ecological niche where heterotrophic and parasitic lifestyles become advantageous^30,114^. Such conditions underscore the need to consider the physical, chemical, and biological interplays, including nutrient influx and light availability, in shaping microbial community compositions.

### Influence of the rare biosphere

To investigate if low abundance taxa were driving the overall diversity patterns, we also conducted beta-diversity analyses on the rare biosphere (Fig. 4). Microbial communities are often dominated by low-abundance taxa^115^, which may be permanently or transiently rare, switch between abundance and rarity, or always be rare with periodic changes in abundance^115,116^. Permanently rare taxa are associated with distinct life history strategies and trade-offs and may occur only in specific ecological niches^116^. The MK sample clustering (Fig. 4) suggested that the diversity of this community was driven more strongly by quantitative (abundance) than by qualitative (presence/absence) differences in rare species. The BL pattern of clustering together with some CB samples in the weighted UniFrac ordination (Fig. 4) suggested that there were quantitative and qualitative similarities to rare taxa in the CB community. However, rare taxa did not drive the structure of the CB community, as the CB samples did not form distinct and exclusive clusters in any of the rare biosphere ordinations (Fig. 4). Both ICE and MK rare biospheres exhibited phylogenetic overdispersion, in contrast to the BL rare biosphere, which was phylogenetically clustered. These suggest that the rare biosphere was a strong driver of phylogenetic community structure in the ICE and MK communities but much less so in the BL and CB communities. Previous research on the AO rare biosphere suggested that it is driven primarily by stochastic processes, in contrast to abundant communities, which were more influenced by deterministic selection^59^. Given that the AO under-ice habitat fluctuates between seasons and years and may require specific physiological adaptations to survive^117^, the dominance of the rare biosphere here is ecologically coherent.

The influence of freshwater may also have played a role in the dominance of the rare biosphere, since the lowest salinities were observed in the Mackenzie River-influenced region, and the under-ice habitat may also have been fresher than surrounding open ocean surface waters. In the Arctic summer, meltwater from the sea ice bottom can accumulate and form a layer of fresher water immediately beneath the sea ice^118^, and riverine inputs also intensify due to increased meltwater and rainfall^119^. Given that both MK and ICE communities are likely to be subjected to both stochastic and seasonal environmental fluctuations and increased freshwater input due to environmental change, these results hint at possible disruption of these AO rare biosphere communities under climate change^120^.

### The role of sea ice in structuring microbial communities

The loss of multi-year ice (MYI) and decreasing duration of the ice-covered period across the Arctic impacts protist communities associated with sea ice^29,121,122^. The ice-water interface beneath first-year ice (FYI) has been found to contain relatively more sea ice protists (but lower protist diversity) than beneath MYI^123^. This could result from increased light penetration to surface waters covered by FYI, which is less thick than MYI. The Western Arctic Ocean is predicted to become dominated by FYI, with an expected increase in primary productivity coinciding with a decrease in ice-associated algal diversity^124^.

In this study, we sampled surface water at the ice-water interface immediately beneath MYI (> 1.5 m thick). The influence of ice was evident by species recovered from samples immediately beneath sea ice, including typical ice-associated species such as *Carteria* spp.^125^ and *Polarella glacialis*^121^. Our phylogenetic approach detected the distinct under-ice community, in keeping with sea ice’s importance in structuring microbial assemblages. This unique ice community was also apparent when looking at heterotroph-only and rare biosphere data subsets, suggesting that these two groups were drivers of the observed structure.

Although diatoms are typically the major primary producers found in sea ice habitats^123,126^, our under-ice samples contained a greater proportional abundance of ciliates and dinoflagellates than diatoms. This could reflect the time of year the samples were taken; there is relatively little known about MYI communities, and light and nutrient limitation at the end of summer could also have affected diatoms more severely. In addition, flagellates may dominate algal communities under low light conditions which may be expected to occur under thick MYI^127^. Diatoms have also been found to contribute little to surface AO communities in late summer, with dinoflagellates being the dominant phylogenetic group^108^; we observed similar patterns in the CB cluster with many dinoflagellate signature ASVs. Overall, the low surface nutrients would have created unfavourable conditions for primary productivity. The dominance of ciliates and dinoflagellates in the under-ice community was also reflected in the signature ASVs, where most species were ciliates or dinoflagellates (Fig. 6e). Previous research on ciliates within and beneath sea ice has suggested that both ciliate diversity and abundance are greater in the water column immediately under the ice than within the ice itself^128^. Alveolates, including ciliates and dinoflagellates, were previously found to be more dominant in the Canada Basin than in the Bering Strait region, where stramenopiles dominated^129^. There were three diatom species among the under-ice signature ASVs, all pennate. Pennate diatoms are more commonly associated with sea ice than centric diatoms^122,123,130,131^, although the low abundance of centric diatoms in our dataset could also be due to our pre-filtering step which removed cells >50 µm; centric diatoms tend to have larger cell sizes than pennate diatoms^132^. Nonetheless, the relative abundance of diatoms at the ice-water interface in our samples was low, but their classification as signature taxa of the samples implies a high level of uniqueness to this community.

Six of the signature ASVs of the under-ice community were assigned as Spirotrichea ciliates, which are obligate mixotrophs^133^. All ciliates are heterotrophic; however, many species can acquire and retain their photosynthetic prey or chloroplasts from their prey and therefore acquire mixotrophy^128^. Spirotrichea ciliates have been commonly found in the Arctic^134^, including in melt ponds associated with sea ice^135^, so their presence in our samples is not unexpected. The top signature ASV (with the highest ASV index value) came from an unclassified ciliate in the clade Phyllopharyngea; species belonging to this group are generally parasitic or prey on other ciliates^136^. The species found in the under-ice community may have been a predator feeding on the abundant ciliates present in these samples. There were also two under-ice signature ASVs classified as *Cryothecomonas aestivalis*, a heterotrophic nanoflagellate commonly associated with sea ice^137^.

Our results highlight not only the spatial heterogeneity of surface AO protist communities but also the contribution of the sea ice habitat to shaping microbial assemblages. Phylogenetic diversity varied by community; the greatest diversity was in the MK community, whilst the lowest diversity, indicative of strong selection pressure, was seen within the ICE community. The greatest mean NRI values were in the BL and CB communities and were indicative of phylogenetic overdispersion and competition-driven community assemblages^53,104^. By contrast, the ICE community had the lowest mean NRI, signifying that phylogenetic clustering and habitat filtering had occurred^53^. The ICE community structure was driven by heterotrophs and rare species, which were more closely related than expected by chance due to their similar ecologies and traits, which allowed them to occupy this environment^104^. This emphasises the degree of ecological specialisation in ice-associated microbial communities and the vulnerability of these communities to ongoing losses in MYI. With the continued and accelerated rates of sea ice loss in the Arctic and the transition from a MYI to a FYI-dominated system, shifts in microbial community assemblages may occur due to changing selection pressures. Phylogenetically selective ice-associated communities composed of rarer species could gradually be replaced with more generalist assemblages and common taxa as the ice habitat is lost, or entirely different assemblages may form. If this happens, AO microbial community structure and function will be altered^68^, with potentially widespread impacts on the wider AO ecosystem.

## Supplementary Information

Supplementary Figures.

Supplementary Tables.

## Data Availability

The sequence data generated and analysed during the study are available at the NCBI Sequence Read Archive (SRA) repository under the accession number PRJNA931954. The ASV sequences, ASV phylogeny, ASV table and taxonomic classification, OTU table and OTU phylogeny are available from the Zenodo repository (doi:105821/zenodo.7612768).
