# Supplementary material for: Vulnerability of Arctic Ocean microbial eukaryotes to sea ice loss
Source: Sci Rep. 2024 Nov 21;14:28896. doi: 10.1038/s41598-024-77821-9 (PMC11582671; doi:10.1038/s41598-024-77821-9)
Supplement: Supplementary file 1 — Supplementary Figures. [file 41598_2024_77821_MOESM1_ESM.pdf]

Supplementary figures

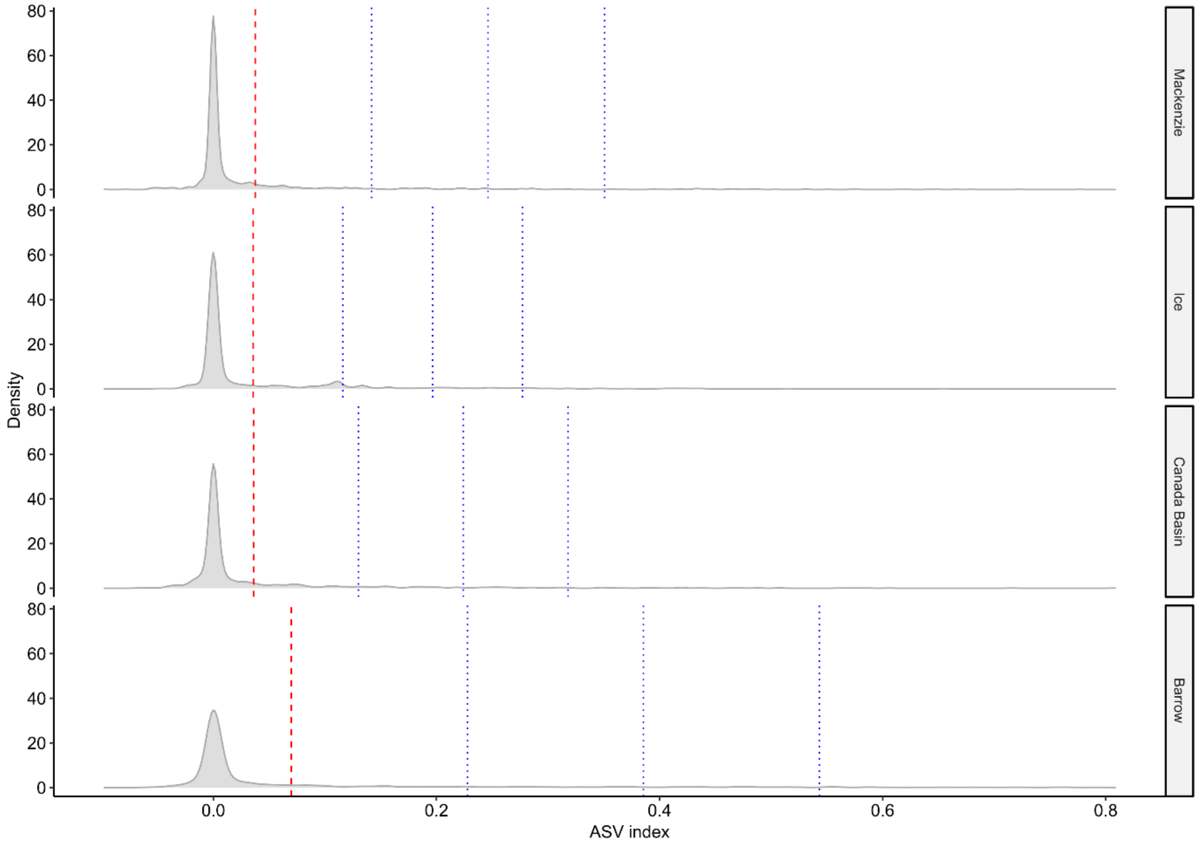

Figure S1. Density distribution plots of ASV index values for each community. Dashed red lines represent the mean values and each dotted blue line represents one standard deviation from the mean.

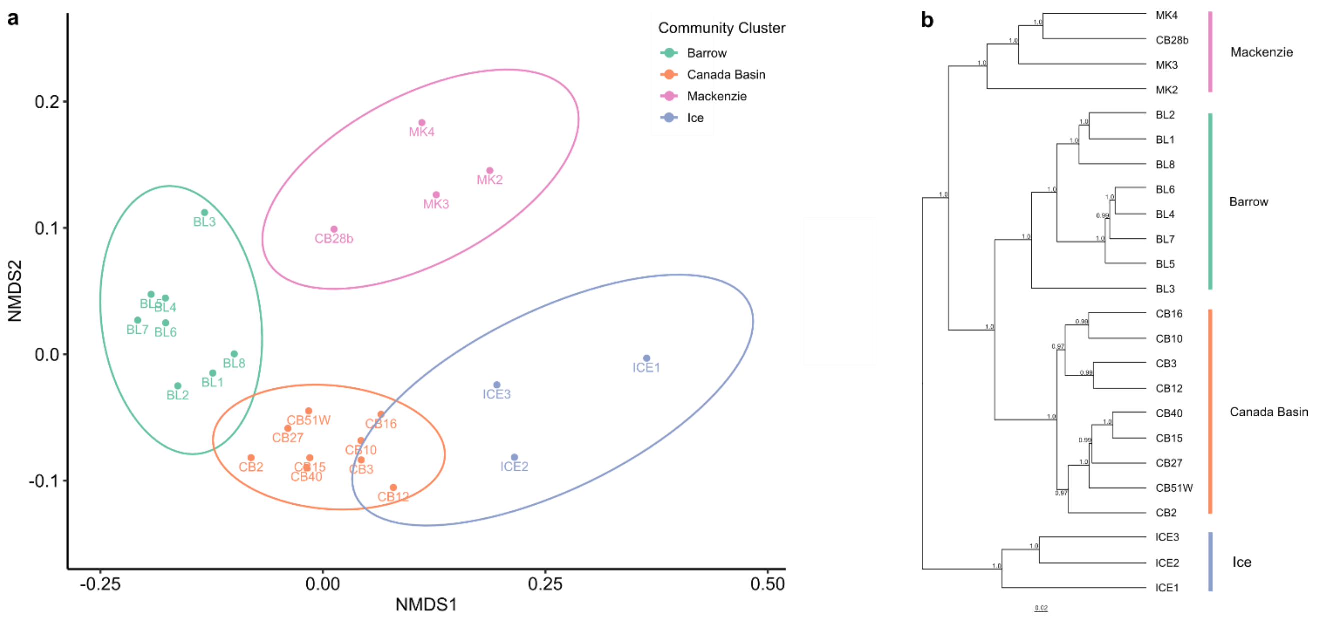

Figure S2. a) NMDS ordination of weighted UniFrac distances based on OTUs clustered at 98% similarity (stress = 0.086). Stations are coloured by clusters which were assigned by UPGMA clustering and ellipses represent 95% confidence intervals around clusters. **b) UPGMA clustering of samples** at a sequence rarefaction depth of 20000 with 1000 iterations.

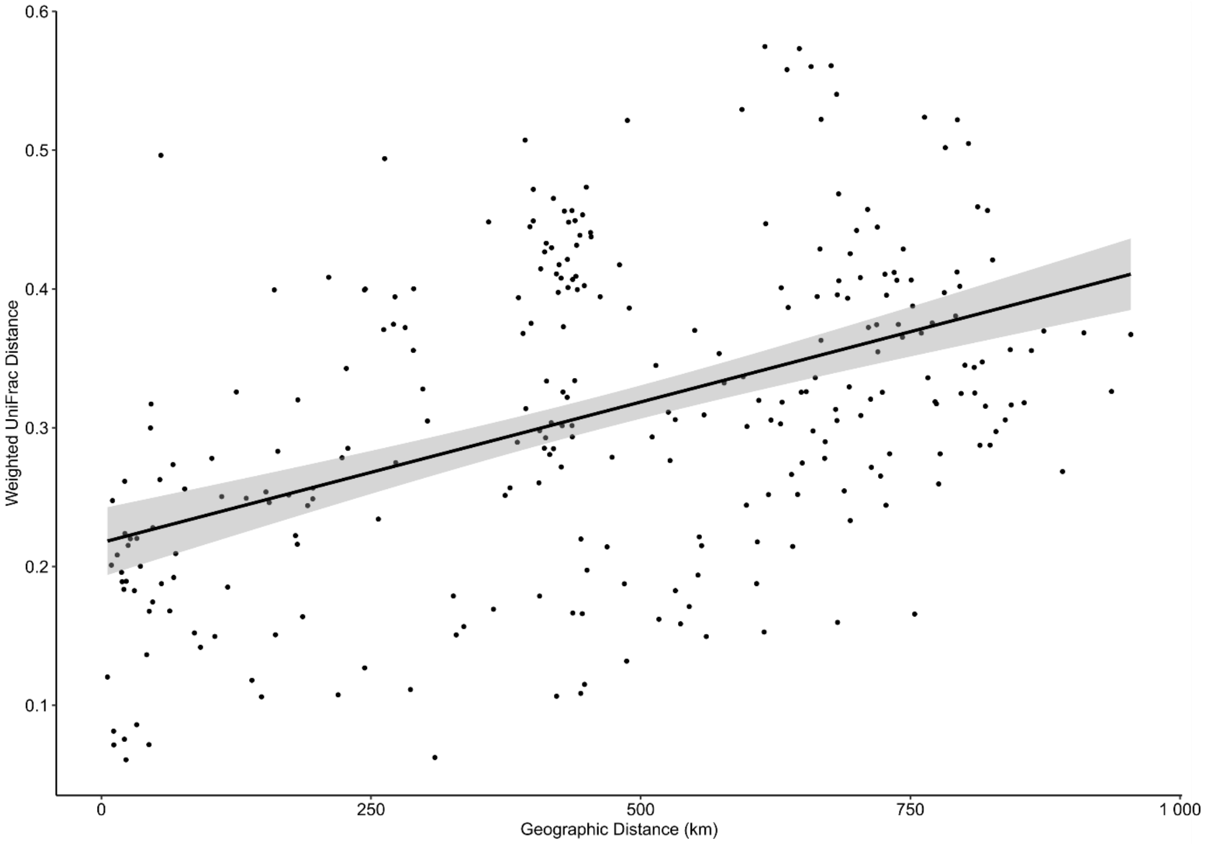

Figure S3. Distance-decay plot. Geographic distances plotted against weighted UniFrac distances between samples, with a distance-decay model (generalised linear model, betapart R package) fitted to the data (pseudo-R^2 = 0.217, p = 0.001).

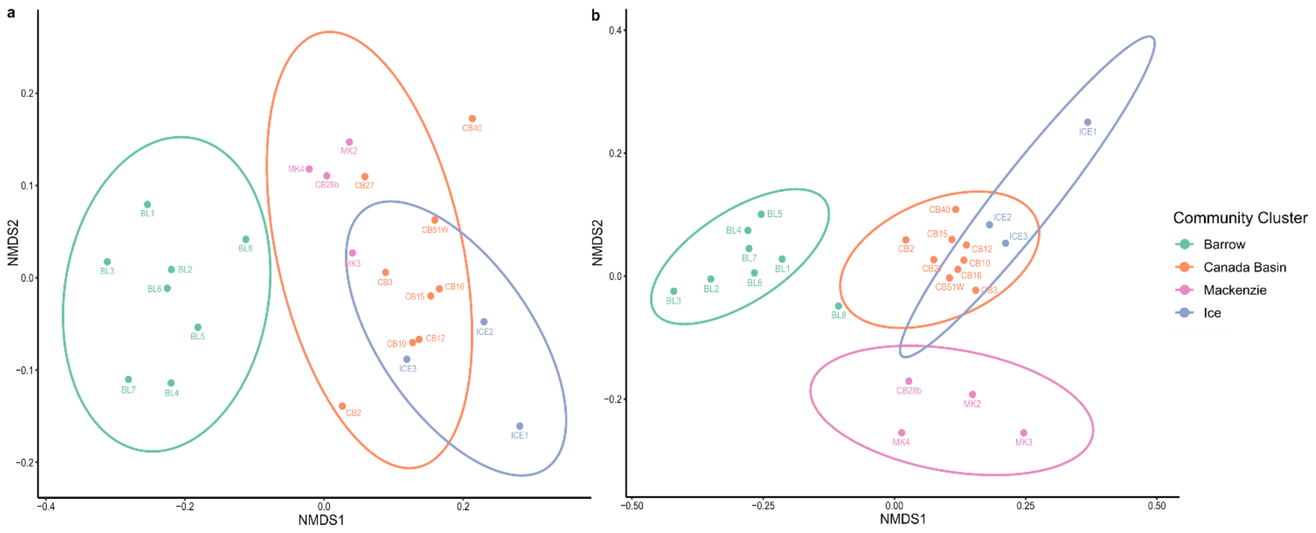

Figure S4. a) NMDS ordination of unweighted UniFrac distances (stress = 0.101). **b) Ordination plot of Bray-Curtis dissimilarities** (stress = 0.110). Stations are coloured by clusters which were assigned by hierarchical clustering and ellipses represent 95% confidence intervals around clusters.

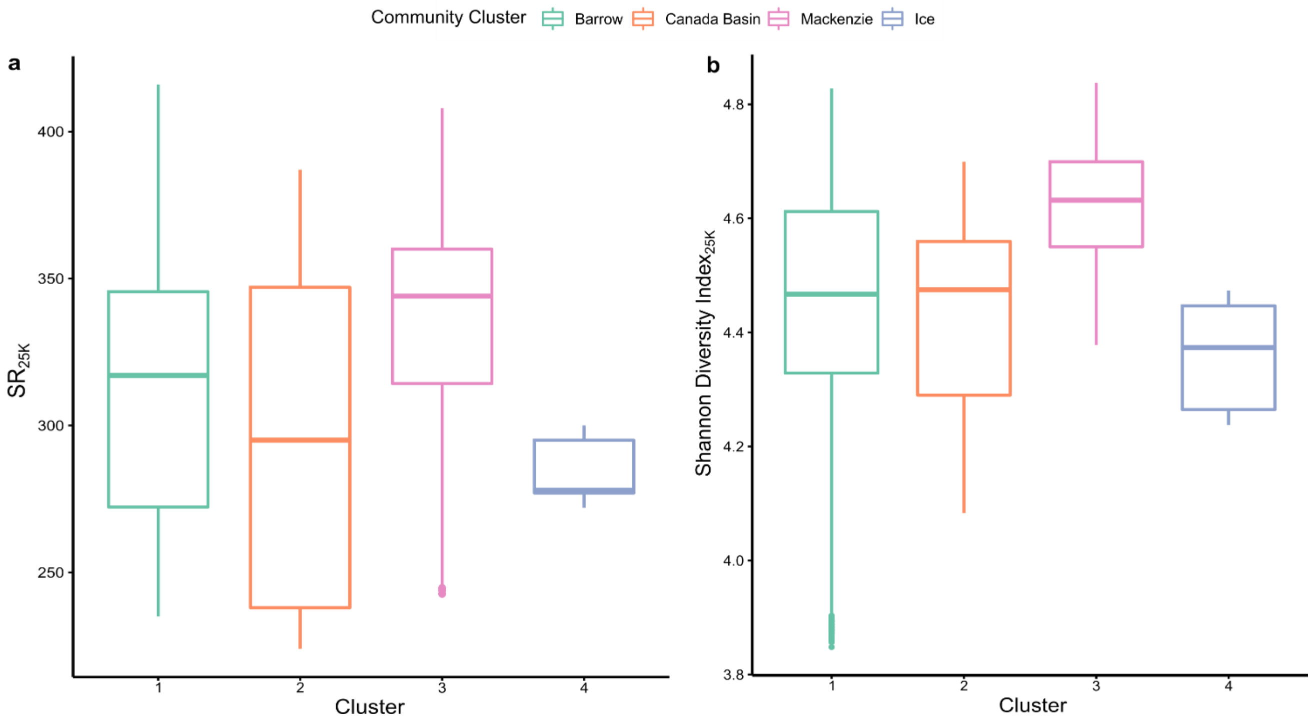

Figure S5. Alpha diversity measures at a sampling depth of 25000 sequences for samples grouped by weighted UniFrac cluster. a) Boxplots of species richness (number of ASVs) observed for each community. **b)** Boxplots of Shannon diversity index values for each community.

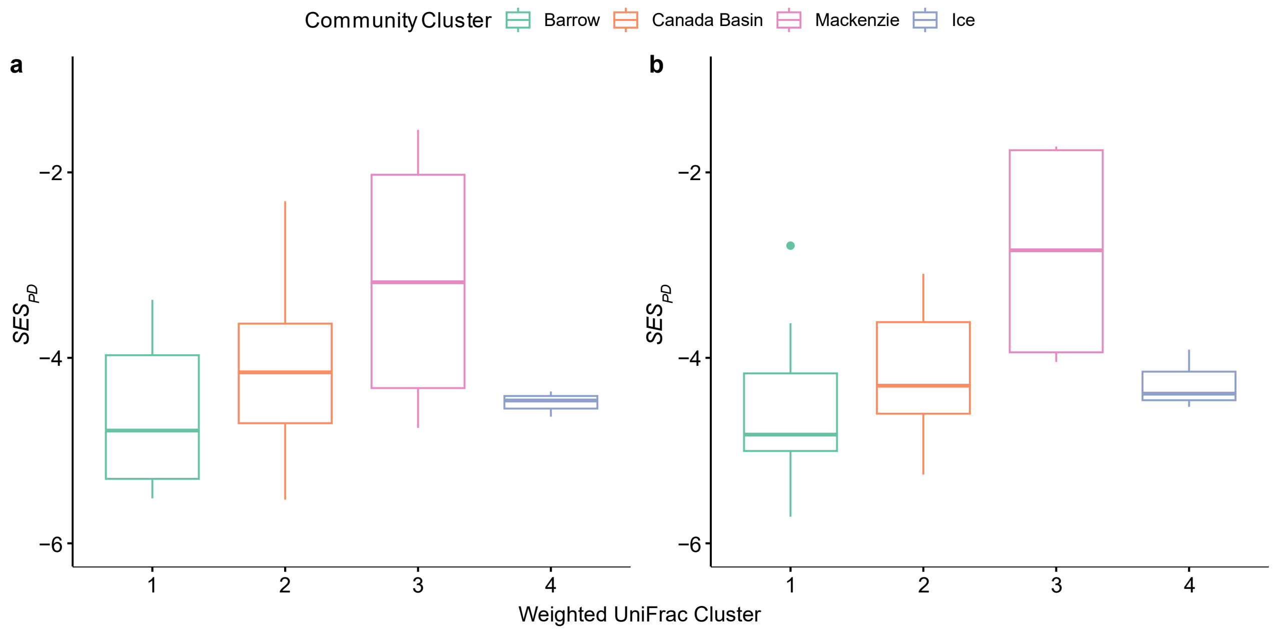

Figure S6. Standardised effect sizes of Faith's phylogenetic diversity (*SES_{PD}*) of each community cluster. a) Boxplots of *SES_{PD}* observed for each community on a rarefied (sampling depth = 25000 sequences) ASV table. **b)** Boxplots of *SES_{PD}* observed for each community on a non-rarefied ASV table.
