# Supplementary material for: Vulnerability of Arctic Ocean microbial eukaryotes to sea ice loss
Source: Sci Rep. 2024 Nov 21;14:28896. doi: 10.1038/s41598-024-77821-9 (PMC11582671; doi:10.1038/s41598-024-77821-9)
Supplement: Supplementary file 2 — Supplementary Tables. [file 41598_2024_77821_MOESM2_ESM.pdf]

JOIS Station	Lat	Long	Temp [ITS-90 C]	Salinity [PSU]	O_2 [mL/L]	SPAR [uE/m2/sec]	NO_3 [mmol/m^3]	SiO_4 [mmol/m^3]	PO_4 [mmol/m^3]	NH_4 [mmol/m^3]	CDOM [mg/m^3]	ChlTOT [ug/L]	PhaeTOT [ug/L]
BL1	71.3627	-152.0780	1.2916	28.8711	8.2225	251.2950	0.0986	5.4644	0.6285	0.2904	3.4411	1.0545	0.3061
BL2	71.3937	-151.9525	1.6882	28.1630	7.9204	128.8900	0.0000	5.4062	0.5180	0.1299	4.1883	1.1433	0.3733
BL3	71.4655	-151.8197	1.2206	28.4121	8.1377	342.0450	0.0000	5.1902	0.5690	0.1759	3.5385	1.1691	0.3812
BL4	71.5210	-151.5867	0.5218	27.5155	8.3623	61.4690	0.0000	4.2865	0.5045	0.0469	3.2865	0.6501	0.2857
*BL5 (BL4)	71.5950	-151.3653	0.5218	27.5155	8.3623	61.4690	0.0000	4.2865	0.5045	0.0469	3.2865	0.6501	0.2857
BL6	71.6817	-151.1867	0.2837	27.3188	8.4061	9.9144	0.0000	5.1546	0.5270	0.0288	3.2793	0.7642	0.2518
*BL7 (BL8)	71.8197	-150.7630	0.1104	26.2459	8.5143	23.7950	0.0000	4.6153	0.4740	0.0194	3.5462	0.6838	0.2094
BL8	71.9547	-150.2595	0.1104	26.2459	8.5143	23.7950	0.0000	4.6153	0.4740	0.0194	3.5462	0.6838	0.2094
CB10	78.3293	-152.3160	-1.4821	27.9285	8.7568	9.9144	0.0000	2.9502	0.5660		1.8258	0.0745	0.0562
CB12	77.6950	-146.6858	-1.3935	27.7248	8.7059	109.0600	0.0000	2.7218	0.5510		1.6903	0.0790	0.0491
CB15	77.0107	-139.9382	-1.5020	28.1920	8.7448	1587.30	0.0000	2.2266	0.5335		1.9994	0.1000	0.0938
CB16	77.9530	-139.9397	-1.5217	28.3786	8.6984	0.0000	0.0000	2.2687	0.5560		2.0643	0.1600	0.1600
CB2	73.0013	-150.0075	-0.9664	25.8166	8.7382	537.0550	0.0000	2.7434	0.4985	0.0039	2.3364	0.0723	0.0687
CB27	72.9977	-140.0003	0.2436	26.2539	8.5449	107.0800	0.0000	2.4629	0.4950		1.2588	0.0365	0.0205
CB28b	70.9983	-139.9958	0.1908	24.8457	8.5882	9.9144	0.0000	3.7761	0.4350	0.0103	2.9065	0.1990	0.1905
CB3	74.0010	-150.0057	-0.1967	26.4406	8.5652	9.9144	0.0000	2.6272	0.5035		1.3245	0.0504	0.0277
CB40	74.4775	-135.3713	-1.4674	27.4815	8.7428	10.0667	0.0000	2.2822	0.5045		1.9407	0.0776	0.0558
CB51W	73.4872	-132.6037	-1.4574	27.3139	8.7885	0.0000	0.0000	2.2060	0.5195		1.7572	0.0701	0.0632
*ICE1 (CB17)	76.5170	-140.0670	-1.3078	27.4600	8.7295	9.9144	0.0000	2.6516	0.5395		1.7457	0.0499	0.0172
*ICE2 (CB16)	78.1280	-140.3580	-1.5276	28.3822	8.6783	0.0000	0.0000	2.2668	0.5420		2.0214	0.1602	0.1585
*ICE3 (CB9)	77.9120	-148.3480	-1.4410	27.7438	8.7690	9.9144	0.0000	2.8525	0.5555		1.8179	0.0637	0.0324
MK2	70.4030	-140.0032	-0.9696	26.5467	8.5876	0.0000	0.0000	2.8595	0.4915	0.0061	2.3876	0.0801	0.0619
MK3	70.5707	-140.0010	-1.0726	26.8164	8.6650	9.9144	0.0000	2.6170	0.4900	0.0020	2.4397	0.0654	0.0563
MK4	70.8115	-140.0015	0.2573	25.3006	8.4889	9.9144	0.0000	3.6062	0.4460	0.0099	2.6328	0.2772	0.2377

Table S1. Environmental metadata table. Measures of environmental data taken at sampling stations. Where there was more than one cast per station and depth from the 2018 CTD data, the mean value was used for geochemical and CTD data. Lat = Sample station latitude; Long = Sample station longitude; O_2 = Mean Downcast Oxygen; SPAR = Surface Reference Photosynthetically Active Radiation; NO_3 = Nitrate and Nitrite; SiO_4; Reactive Silicate; PO_4 = Orthophosphate; CDOM = Coloured Dissolved Organic Matter; ChlTOT = Total Chlorophyll (0.7µm filter); PhaeTOT = Total Phaeopigments (0.7µm filter).

* Denotes stations for which environmental metadata came from measurements taken at the nearest adjacent station (indicated in parentheses). Latitudes and longitudes refer to the locations of sampling, not metadata measures.

Table S2. BL signature ASVs. Table listing the ASVs that were found to have ASV index values greater than three standard deviations above the mean value (0.543) in the BL community. ASVs are ordered from high to low ASV index, and each ASV's relative abundance in the community is also shown for reference.

ASV ID	Supergroup	Division	Class	Species	ASV Index	Relative Abundance
ASV_42	Alveolata	Ciliophora	Spirotrichea	Leegaardiella_sp.	0.800213692	0.0161
ASV_539	Hacrobia	Haptophyta	Haptophyta_Clade_HAP3	Haptophyta_Clade_HAP3_XXX_sp.	0.791984586	0.000353957
ASV_444	Archaeplastida	Chlorophyta	Mamiellophyceae	Micromonas_clade_B3	0.782612227	0.000706348
ASV_81	Stramenopiles	Ochrophyta	Pelagophyceae	Aureococcus_anophagefferens	0.768956679	0.006267528
ASV_65	Hacrobia	Telonemia	Telonemia_X	Telonemia-Group-1_X_sp.	0.763170735	0.000276396
ASV_158	Hacrobia	Haptophyta	Prymnesiophyceae	Prymnesiophyceae_Clade_E_XX_sp.	0.762316073	0.004273423
ASV_306	Stramenopiles	Sagenista	MAST-7	MAST-7B_XX_sp.	0.756111972	0.000683793
ASV_68	Archaeplastida	Chlorophyta	Pyramimonadales	Pyramimonas_australis	0.754777839	0.011485562
ASV_184	Rhizaria	Cercozoa	Filosa-Thecofilosea	Protaspa-lineage_X_sp.	0.74225615	0.001980582
ASV_216	Alveolata	Ciliophora	Spirotrichea	Strombidium_caudispina	0.728607936	0.001837115
ASV_252	Hacrobia	Cryptophyta	Cryptophyceae	Hemiselmis_cryptochromatica	0.725383689	0.00201757
ASV_15	Hacrobia	Haptophyta	Prymnesiophyceae	Phaeocystis_pouchetii	0.719353571	0.040144114
ASV_311	Hacrobia	Haptophyta	Prymnesiophyceae	Prymnesiophyceae_Clade_B3_X_sp.	0.713705191	0.001354456
ASV_28	Hacrobia	Telonemia	Telonemia_X	Telonemia-Group-2_X_sp.	0.706479997	0.042745274
ASV_660	Hacrobia	Telonemia	Telonemia_X	Telonemia-Group-2_X_sp.	0.695511632	0.000188147
ASV_722	Stramenopiles	Ochrophyta	Bacillariophyta	Eucampia_sp.	0.695113727	0.000130903
ASV_478	Stramenopiles	Ochrophyta	Dictyochophyceae	Florenciella_parvula	0.682171222	0.000557991
ASV_106	Hacrobia	Telonemia	Telonemia_X	Telonemia-Group-2_X_sp.	0.67988972	0.008050016
ASV_110	Stramenopiles	Ochrophyta	Bolidophyceae	Parmales_env_1_X_sp.	0.671958959	0.005574325
ASV_96	Stramenopiles	Ochrophyta	Bacillariophyta	Minidiscus_trioculatus	0.66439162	0.004339877
ASV_456	Rhizaria	Cercozoa	Filosa-Thecofilosea	Cryothecomonas_aestivalis	0.657523483	0.000671848
ASV_317	Rhizaria	Cercozoa	Filosa-Thecofilosea	Ebria_tripartita	0.65171286	0.00063832
ASV_164	Hacrobia	Haptophyta	Pavlovophyceae	NA	0.64932867	0.004226919
ASV_412	Rhizaria	Cercozoa	Filosa-Imbricatea	Novel-clade-2_X_sp.	0.637801293	0.000860107
ASV_364	Rhizaria	Cercozoa	Filosa-Imbricatea	Paulinella_sp.	0.631948208	0.000617813
ASV_525	Alveolata	Dinoflagellata	Dinophyceae	Pelagodinium_beii	0.625025124	0.000444845
ASV_501	Alveolata	Ciliophora	Spirotrichea	Helicostomella_subulata	0.610840055	0.000580042
ASV_498	Hacrobia	Haptophyta	Prymnesiophyceae	Chrysochromulina_sp.	0.610222439	0.000337229
ASV_93	Alveolata	Ciliophora	Spirotrichea	Leegaardiella_sp.	0.598366171	0.013640837
ASV_189	Hacrobia	Picozoa	Picozoa_X	Picozoa_XXXX_sp.	0.591896691	0.002099198

ASV_105	Hacrobia	Haptophyta	Prymnesiophyceae	Phaeocystis_sp.	0.590088772	0.010599394
ASV_653	Stramenopiles	Ochrophyta	Pelagophyceae	NA	0.586261608	0.000276396
ASV_432	Alveolata	Ciliophora	CONThreeP	Askenasia_sp.	0.584731051	0.0008327
ASV_440	Rhizaria	Radiolaria	Acantharea	Acantharea_XXX_sp.	0.582626407	0.000726079
ASV_641	Alveolata	Dinoflagellata	Syndiniales	Dino-Group-II-Clade-6_X_sp.	0.565867821	8.49E-05
ASV_352	Hacrobia	Haptophyta	Prymnesiophyceae	Chrysochromulina_sp.	0.559737276	0.001436097
ASV_415	Opisthokonta	Fungi	Fungi_X	Fungi_XXXX_sp.	0.558421998	0.000165358
ASV_9	Alveolata	Dinoflagellata	Dinophyceae	Heterocapsa_pygmaea	0.556774328	0.088151155
ASV_102	Alveolata	Ciliophora	Spirotrichea	Strombidiidae_H_X_sp.	0.555769518	0.007880754
ASV_8	Archaeplastida	Chlorophyta	Pyramimonadales	Pyramimonadales_XXX_sp.	0.555553559	0.029948337
ASV_388	Alveolata	Dinoflagellata	Dinophyceae	Heterocapsa_nei/rotundata	0.553205904	0.000448716
ASV_632	Alveolata	Dinoflagellata	Syndiniales	Dino-Group-II-Clade-1_X_sp.	0.551152054	0.000226929
ASV_39	Archaeplastida	Chlorophyta	Mamiellophyceae	Micromonas_commoda_A2	0.55024063	0.008365891
ASV_221	Alveolata	Ciliophora	Spirotrichea	Leegaardiella_sp.	0.545171439	0.001232582

Table S3. CB signature ASVs. Table listing the ASVs that were found to have ASV index values greater than three standard deviations above the mean value (0.318) in the CB community. ASVs are ordered from high to low ASV index, and each ASV's relative abundance in the community is also shown for reference.

ASV ID	Supergroup	Division	Class	Species	ASV Index	Relative Abundance
ASV_370	Archaeplastida	Chlorophyta	Mamiellophyceae	Mantoniella_squamata	0.808492577	0.000546
ASV_257	Alveolata	Dinoflagellata	Syndiniales	Dino-Group-III_XX_sp.	0.715776336	0.001217
ASV_579	Rhizaria	Cercozoa	Filosa-Thecofilosea	Filosa-Thecofilosea_XXX_sp.	0.605424338	0.000267
ASV_289	Alveolata	Dinoflagellata	Dinophyceae	Prorocentrum_cordatum	0.58665829	0.001311
ASV_416	NA	NA	NA	NA	0.577912261	0.000581
ASV_678	Alveolata	Ciliophora	Phyllopharyngea	NA	0.569436319	0.000197
ASV_210	Alveolata	Dinoflagellata	Dinophyceae	Dinophyceae_XXX_sp.	0.560543606	0.001286
ASV_137	Alveolata	Dinoflagellata	Syndiniales	Dino-Group-III_XX_sp.	0.508859737	0.003005
ASV_428	Alveolata	Dinoflagellata	Dinophyceae	NA	0.506249156	0.000448
ASV_236	Alveolata	Dinoflagellata	Dinophyceae	Prorocentrum_sp.	0.488651633	0.001193
ASV_548	Alveolata	Dinoflagellata	Dinophyceae	Dinophyceae_XXX_sp.	0.486981702	0.000132
ASV_580	Alveolata	Dinoflagellata	Syndiniales	Dino-Group-III_XX_sp.	0.473910439	0.000176
ASV_422	Rhizaria	Cercozoa	Filosa-Thecofilosea	Ventricleftida_XX_sp.	0.467437005	0.000528
ASV_635	Alveolata	Dinoflagellata	Dinophyceae	NA	0.461488509	0.000212
ASV_323	Alveolata	Dinoflagellata	Dinophyceae	Dinophyceae_XXX_sp.	0.441403938	0.000467
ASV_239	Alveolata	Dinoflagellata	Syndiniales	Dino-Group-I-Clade-1_X_sp.	0.434868078	0.000861
ASV_645	Apusozoa	Apusomonadidae	Apusomonadidae_Group-1	Apusomonadidae_Group-1_XXX_sp.	0.422404937	0.00018
ASV_3	Alveolata	Dinoflagellata	Dinophyceae	Gymnodinium_sp.	0.422207273	0.060005
ASV_377	Alveolata	Dinoflagellata	Dinophyceae	NA	0.417284227	0.000771
ASV_86	Alveolata	Dinoflagellata	Dinophyceae	Prorocentrum_sp.	0.41663224	0.005187
ASV_253	Alveolata	Dinoflagellata	Dinophyceae	Gyrodinium_rubrum	0.404485308	0.001437
ASV_282	Alveolata	Dinoflagellata	Dinophyceae	NA	0.395278106	0.00098
ASV_30	Alveolata	Dinoflagellata	Dinophyceae	Prorocentrum_sp.	0.393402385	0.010759
ASV_31	Alveolata	Ciliophora	Spirotrichea	Strombidiidae_H_X_sp.	0.387349233	0.007764
ASV_120	Alveolata	Dinoflagellata	Dinophyceae	NA	0.386154696	0.002592
ASV_208	Alveolata	Dinoflagellata	Dinophyceae	Gymnodinium_sp.	0.370343842	0.001288
ASV_151	Alveolata	Dinoflagellata	Dinophyceae	NA	0.361670585	0.002519
ASV_344	Stramenopiles	Sagenista	MAST-9	MAST-9D_XX_sp.	0.354789674	0.000736
ASV_700	Alveolata	Dinoflagellata	Dinophyceae	NA	0.353068463	9.25E-05
ASV_147	Alveolata	Dinoflagellata	Syndiniales	Dino-Group-I-Clade-1_X_sp.	0.347768672	0.001644

ASV_100	Alveolata	Dinoflagellata	Dinophyceae	NA	0.346975756	0.003438
ASV_397	Stramenopiles	Ochrophyta	Dictyochophyceae	Dictyocha_speculum	0.34497335	0.000298
ASV_49	Hacrobia	Haptophyta	Prymnesiophyceae	Phaeocystis_sp.	0.320935876	0.005739

Table S4. MK signature ASVs. Table listing the ASVs that were found to have ASV index values greater than three standard deviations above the mean value (0.351) in the MK community. ASVs are ordered from high to low ASV index, and each ASV's relative abundance in the community is also shown for reference.

ASV ID	Supergroup	Division	Class	Species	ASV Index	Relative Abundance
ASV_103	Alveolata	Ciliophora	Phyllopharyngea	PHYLL_4_X_sp.	0.775811	0.011568
ASV_192	Rhizaria	Cercozoa	Filosa-Thecofilosea	NA	0.722205	0.002421
ASV_218	Hacrobia	Haptophyta	Prymnesiophyceae	Chrysochromulina_sp.	0.678883	0.002421
ASV_78	Hacrobia	Haptophyta	Prymnesiophyceae	Chrysochromulina_sp.	0.665479	0.009939
ASV_56	Alveolata	Ciliophora	Spirotrichea	NA	0.64932	0.013735
ASV_60	Stramenopiles	Ochrophyta	Chrysophyceae	Chrysophyceae_Clade-C_X_sp.	0.641844	0.03331
ASV_688	Rhizaria	Cercozoa	Cercozoa_X	Cercozoa_XXXX_sp.	0.62193	0.00028
ASV_568	Alveolata	Dinoflagellata	Dinophyceae	Protoperidinium_sp.	0.614117	0.000439
ASV_144	Stramenopiles	Ochrophyta	Bacillariophyta	Chaetoceros_lorenzianus_2	0.57818	0.007055
ASV_395	Stramenopiles	Ochrophyta	Bacillariophyta	Thalassionema_sp.	0.572212	0.001021
ASV_448	Alveolata	Ciliophora	Spirotrichea	Strobilidiidae_A_X_sp.	0.551337	0.00029
ASV_301	Alveolata	Ciliophora	CONThreeP	Urotricha_sp.	0.5315	0.002365
ASV_126	Alveolata	Dinoflagellata	Syndiniales	Dino-Group-II_XX_sp.	0.531095	0.014957
ASV_611	Stramenopiles	Ochrophyta	Bacillariophyta	Chaetoceros_decipiens	0.503369	0.000299
ASV_162	Alveolata	Ciliophora	Litostomatea	Pleurostomatida_X_sp.	0.488212	0.004684
ASV_329	Alveolata	Ciliophora	Oligohymenophorea	OLIGO5_XX_sp.	0.486286	0.001426
ASV_319	Alveolata	Dinoflagellata	Syndiniales	Dino-Group-II-Clade-16_X_sp.	0.484335	0.002902
ASV_813	Stramenopiles	Pseudofungi	Oomycota	NA	0.479228	0.000166
ASV_70	Stramenopiles	Pseudofungi	MAST-1	MAST-1A_XX_sp.	0.472693	0.020077
ASV_837	Alveolata	Dinoflagellata	Syndiniales	Dino-Group-II-Clade-7_X_sp.	0.467509	0.000171
ASV_308	Alveolata	Dinoflagellata	Syndiniales	NA	0.466345	0.001039
ASV_161	Rhizaria	Radiolaria	Acantharea	Acantharea_XXX_sp.	0.464561	0.005012
ASV_46	Hacrobia	Haptophyta	Prymnesiophyceae	Prymnesiophyceae_Clade_B4_X_sp.	0.46036	0.018169
ASV_80	Alveolata	Ciliophora	Spirotrichea	Strombidiidae_H_X_sp.	0.448042	0.00652
ASV_431	Rhizaria	Radiolaria	Acantharea	Acantharea_XXX_sp.	0.44608	0.000507
ASV_908	Alveolata	Ciliophora	Spirotrichea	NA	0.444064	9.47E-05
ASV_895	Stramenopiles	Ochrophyta	Chrysophyceae	Chrysophyceae_Clade-H_X_sp.	0.440813	0.000111
ASV_127	Alveolata	Ciliophora	Spirotrichea	Strombidium_R_sp.	0.438624	0.009606
ASV_815	Opisthokonta	Choanoflagellida	Choanoflagellatea	Diaphanoeca_grandis	0.435261	0.000142
ASV_644	Opisthokonta	Choanoflagellida	Choanoflagellatea	Diaphanoeca_grandis	0.433575	0.000287

ASV_860	Stramenopiles	Sagenista	MAST-6	MAST-6_XXX_sp.	0.433028	0.00011
ASV_1000	Rhizaria	Cercozoa	NA	NA	0.432293	7.42E-05
ASV_206	Alveolata	Ciliophora	Spirotrichea	Strobilidiidae_A_X_sp.	0.428329	0.002402
ASV_617	Alveolata	Ciliophora	Phyllopharyngea	Chlamydonellopsis_sp.	0.419458	0.000396
ASV_182	Stramenopiles	Ochrophyta	Bacillariophyta	Chaetoceros_rostratus	0.418603	0.005981
ASV_828	Rhizaria	Cercozoa	Filosa-Thecofilosea	CCW10-lineage_X_sp.	0.414468	0.000135
ASV_806	Alveolata	Ciliophora	CONThreeP	Askenasia_sp.	0.404022	0.000154
ASV_391	Alveolata	Ciliophora	CONThreeP	Askenasia_sp.	0.396396	0.001062
ASV_556	Rhizaria	Cercozoa	Filosa-Imbricatea	Filosa-Imbricatea_XXX_sp.	0.392302	0.000348
ASV_590	Alveolata	Dinoflagellata	Syndiniales	Dino-Group-II-Clade-10-and-11_X_sp.	0.368855	0.00065
ASV_703	Rhizaria	Cercozoa	Filosa-Thecofilosea	Ventricleftida_XX_sp.	0.359412	0.000197
ASV_727	Alveolata	Dinoflagellata	Syndiniales	Dino-Group-I-Clade-4_X_sp.	0.354501	0.000111

Table S5. ICE signature ASVs. Table listing the ASVs that were found to have ASV index values greater than three standard deviations above the mean value (0.277) in the ICE community. ASVs are ordered from high to low ASV index, and each ASV's relative abundance in the community is also shown for reference.

ASV ID	Supergroup	Division	Class	Species	ASV Index	Relative abundance
ASV_214	Alveolata	Ciliophora	Phyllopharyngea	PHYLL_4_X_sp.	0.654083445	0.002103
ASV_658	Stramenopiles	Ochrophyta	Bacillariophyta	Raphid-pennate_X_sp.	0.514440015	0.000635
ASV_33	Alveolata	Ciliophora	Litostomatea	Didiniidae_X_sp.	0.440045469	0.095397
ASV_225	Alveolata	Dinoflagellata	Dinophyceae	Chytriodinium_roseum	0.43482343	0.002979
ASV_51	Stramenopiles	Ochrophyta	Bolidophyceae	Parmales_env_1_X_sp.	0.433624809	0.003825
ASV_275	Alveolata	Ciliophora	Litostomatea	Didiniidae_X_sp.	0.426374486	0.002012
ASV_402	Alveolata	Ciliophora	Oligohymenophorea	Philasterida_X_sp.	0.423088367	0.001836
ASV_910	Alveolata	Ciliophora	NA	NA	0.419977499	0.000192
ASV_226	Stramenopiles	Ochrophyta	Chrysophyceae	Dinobryon_faculiferum	0.415678969	0.002207
ASV_398	Alveolata	Ciliophora	Litostomatea	Didiniidae_X_sp.	0.412766555	0.002963
ASV_27	Alveolata	Ciliophora	Phyllopharyngea	PHYLL_4_X_sp.	0.410581375	0.01397
ASV_234	Alveolata	Dinoflagellata	Dinophyceae	NA	0.404875156	0.000286
ASV_219	Alveolata	Dinoflagellata	Dinophyceae	Apocalathium_aciculiferum	0.4036227	0.005693
ASV_544	Alveolata	Dinoflagellata	Dinophyceae	Islandinium_tricingulatum	0.375853291	0.000259
ASV_414	Alveolata	Dinoflagellata	Dinophyceae	Polarella_glacialis	0.369633514	0.001138
ASV_197	Rhizaria	Cercozoa	Filosa-Thecofilosea	Cryothecomonas_aestivalis	0.359626704	0.002634
ASV_471	Alveolata	Ciliophora	Spirotrichea	Hypotrichia_XX_sp.	0.351338614	0.000684
ASV_87	Alveolata	Ciliophora	Phyllopharyngea	PHYLL_4_X_sp.	0.346818912	0.003495
ASV_34	Archaeplastida	Chlorophyta	Chlorophyceae	Carteria_sp.	0.344129844	0.031865
ASV_1141	Alveolata	Ciliophora	Litostomatea	Didiniidae_X_sp.	0.342941964	5.86E-05
ASV_403	Alveolata	Ciliophora	Spirotrichea	Leegaardiellidae_A_X_sp.	0.320388494	0.000311
ASV_325	Stramenopiles	Ochrophyta	Bacillariophyta	Naviculales_sp.	0.319202612	0.001539
ASV_320	Alveolata	Ciliophora	Oligohymenophorea	Peniculida_X_sp.	0.316174474	0.005035
ASV_134	Alveolata	Ciliophora	Spirotrichea	Strombidiidae_H_X_sp.	0.305537228	0.004145
ASV_243	Alveolata	Dinoflagellata	Dinophyceae	NA	0.301879479	0.001328
ASV_296	Alveolata	Dinoflagellata	Syndiniales	Dino-Group-III_XX_sp.	0.29779288	0.000668
ASV_140	Rhizaria	Cercozoa	Filosa-Thecofilosea	Cryothecomonas_aestivalis	0.292525534	0.005345
ASV_864	Alveolata	Ciliophora	Spirotrichea	Euplotes_sp.	0.28691736	0.000266
ASV_720	Alveolata	Ciliophora	Spirotrichea	Rimostrombidium_A_sp.	0.286074635	0.000505
ASV_460	Stramenopiles	Ochrophyta	Bacillariophyta	Bacillaria_paxillifer	0.282979811	0.000296

ASV_177	Stramenopiles	Pseudofungi	MAST-1	MAST-1C_XX_sp.	0.282451231	0.013873
ASV_409	Alveolata	Ciliophora	Spirotrichea	Strombidiidae_R_X_sp.	0.282350766	0.000997
ASV_222	Stramenopiles	Ochrophyta	Bolidophyceae	Parmales_env_1_X_sp.	0.280532267	0.000365
ASV_315	Alveolata	Ciliophora	Litostomatea	NA	0.279351793	0.00369
